# Localizing Epileptic Foci Using Simultaneous EEG-fMRI Recording: Template Component Cross-Correlation

**DOI:** 10.3389/fneur.2021.695997

**Published:** 2021-11-15

**Authors:** Elias Ebrahimzadeh, Mohammad Shams, Masoud Seraji, Seyyed Mostafa Sadjadi, Lila Rajabion, Hamid Soltanian-Zadeh

**Affiliations:** ^1^CIPCE, School of Electrical and Computer Engineering, College of Engineering, University of Tehran, Tehran, Iran; ^2^School of Cognitive Sciences, Institute for Research in Fundamental Sciences (IPM), Tehran, Iran; ^3^Neural Engineering Laboratory, Department of Electrical and Computer Engineering, George Mason University, Fairfax, VA, United States; ^4^Center for Molecular and Behavioral Neuroscience, Rutgers University, Newark, NJ, United States; ^5^Behavioral and Neural Sciences Graduate Program, Rutgers University, Newark, NJ, United States; ^6^School of Graduate Studies, SUNY Empire State College, Manhattan, NY, United States; ^7^Image Analysis Laboratory, Departments of Radiology and Research Administration, Henry Ford Health System, Detroit, MI, United States

**Keywords:** simultaneous EEG-fMRI, epileptogenic zone, independent component analysis (ICA), generalized linear model (GLM), blood-oxygen-level dependent imaging (BOLD), epilepsy, source localization

## Abstract

Conventional EEG-fMRI methods have been proven to be of limited use in the sense that they cannot reveal the information existing in between the spikes. To resolve this issue, the current study obtains the epileptic components time series detected on EEG and uses them to fit the Generalized Linear Model (GLM), as a substitution for classical regressors. This approach allows for a more precise localization, and equally importantly, the prediction of the future behavior of the epileptic generators. The proposed method approaches the localization process in the component domain, rather than the electrode domain (EEG), and localizes the generators through investigating the spatial correlation between the candidate components and the spike template, as well as the medical records of the patient. To evaluate the contribution of EEG-fMRI and concordance between fMRI and EEG, this method was applied on the data of 30 patients with refractory epilepsy. The results demonstrated the significant numbers of 29 and 24 for concordance and contribution, respectively, which mark improvement as compared to the existing literature. This study also shows that while conventional methods often fail to properly localize the epileptogenic zones in deep brain structures, the proposed method can be of particular use. For further evaluation, the concordance level between IED-related BOLD clusters and Seizure Onset Zone (SOZ) has been quantitatively investigated by measuring the distance between IED/SOZ locations and the BOLD clusters in all patients. The results showed the superiority of the proposed method in delineating the spike-generating network compared to conventional EEG-fMRI approaches. In all, the proposed method goes beyond the conventional methods by breaking the dependency on spikes and using the outside-the-scanner spike templates and the selected components, achieving an accuracy of 97%. Doing so, this method contributes to improving the yield of EEG-fMRI and creates a more realistic perception of the neural behavior of epileptic generators which is almost without precedent in the literature.

## Highlights

- In this study, we succeeded in diminishing limitations through presenting a method in the component domain for localizing epileptic foci, taking into account the clinical application, so that more satisfactory results than the conventional EEG-fMRI methods could be obtained.
- The component-based method plays a more prominent role in eliminating the need for invasive electrode implantations compared to conventional EEG-fMRI analysis.
- The component-based method brings to attention the variations in amplitude and duration of epileptic spikes, whereas the conventional methods simplistically assume that all events are equal.
- The conventional approach overlooks the fact that IED activity is continuous and contains fluctuating sub-threshold epileptic activity that is not clearly observed on surface EEG recordings.
- Such valuable information will be obtained by the ICA algorithm applied as part of the proposed method.

## Introduction

Epilepsy is one of the most common neurological disease worldwide (1). It is generally characterized by an enduring predisposition to recurrent yet spontaneous seizures, defined as brief episodes of signs or symptoms indicating excessive, abnormal, or synchronous neuronal activity in the brain (2). The first course of treatment for this condition is drug therapy. However, about 30% of patients are refractory to antiepileptic medications (2), and those with focal epilepsy may be considered for epilepsy surgery.

To provide successful surgical treatment, an improved preoperative evaluation that delineates the epileptogenic zone (EZ) is a critical prerequisite. Several methods have been proposed in the literature (3–5), among which intracranial electroencephalography recording (icEEG) has gained the most attention and is known as the gold standard for defining the epileptogenic zone (EZ) and localizing the seizure onset zone (SOZ) (6). Although popular, this invasive monitoring technique is not without risks or shortcomings (7): it explores only a small fraction of the brain and tends to be time-consuming as the frequency of seizure occurrence is relatively low compared with interictal epileptiform discharges (IEDs). Consequently, over the past few years, greater attention has been directed toward noninvasive EEG-correlated functional magnetic resonance imaging (EEG-fMRI) method as an additional tool to localize the SOZ (8–11). EEG-fMRI combines the high spatial resolution of blood oxygen level-dependent (BOLD) MRI with the high temporal resolution of the EEG signal. This method is now increasingly available following the resolution of crucial technical challenges such as developing suitable amplifiers and procedures for correcting the scanner-related artifacts in the EEG signal (12–15). There is a clinical need for optimized mapping of the changes in neuronal activity related to epileptic discharges observed on surface EEG (16) considering the subclinical nature of some of the interictal epileptiform activity which makes the events of interest only recognizable on the EEG record. Studying the correlation of these events with the fMRI time series reveals complex patterns of hemodynamic change indicative of brain networks. Studies investigating the spike-related BOLD changes have shown that in addition to characterizing different types of focal and generalized epilepsy, these measures could also improve the presurgical evaluation of patients with refractory focal seizures (17–19).

In epilepsy patients, spike-related BOLD changes can contribute to the localization of the epileptic foci. As shown in the literature, the BOLD signal tends to increase in regions that generate spikes (20), although it is often in the form of widespread responses (21). The study of (22) reports a noticeable rate of 60% in seizure freedom in patients who underwent surgical resections where the cortical tissues responsible for the highest spike-correlated BOLD changes were completely removed. Furthermore, the simultaneous recording and analysis of EEG-fMRI is now an important tool in localizing epileptic generators in patients with nonlesional frontal lobe epilepsy, as confirmed by other imaging modalities (19). The literature has found this technique to be of great value when it comes to clinical decision makings. Pittau et al. (18) demonstrated that EEG-fMRI analysis facilitated the localization of epileptic generators in 64% of the patients and the BOLD responses were concordant with the spike-generating regions in 88% of the patients. In patients who were considered ineligible for surgery according to the conventional clinical decision makings, EEG-fMRI confirmed multifocality in 4 of 5 presumed multifocal patients and improved SOZ localization in 4 of 6 patients with unclear foci (23).

According to the conventional method, the IEDs are considered the primary indicators of epileptic activity (24). So, the conventional analysis begins with identifying and marking the IEDs by trained experts assessing the simultaneous EEG-fMRI. The timing of the detected IEDs is then taken as simple epileptic events and convolved with the hemodynamic response function (HRF) to produce a regressor for a General Linear Model (GLM) analysis. Finally, the estimated activation area with the highest statistical significance will be considered as the spike onset zone, a potential marker of the epileptogenic zone (EZ). Many of the recent studies are still based on the GLM analysis (8–15) and their improvement is in increasing of magnetic field strength (25) or using simultaneous intracranial EEG-fMRI (iEEG-fMRI) (26, 27). Yet, the clinical utility of the conventional EEG-fMRI approach is not completely supported by the published literature (9–13). An important limitation of the conventional IED-based EEG-fMRI analysis is that it only considers the information of epileptic focus activity at the time of the spikes and ignores all the neural activities associated with epileptic generators in other time points. So, the actual neural behavior of epileptic generators over the entirety of the recording is not captured by the conventional method, which is unable to localize the epileptic generator when the spikes do not occur during the EEG recording. The proposed method covers this limitation by considering epileptic neural activity regardless of whether or not a spike occurs, potentially leading to a more accurate localization of the epileptogenic zone.

To improve upon the conventional analysis method, we introduced a new method for analyzing EEG-fMRI data that utilizes the information contained within the entire time series of a relevant EEG source. To do this, we first separated the EEG components using independent component analysis (ICA) and calculated the cross-correlation between the time series of the extracted ICA components and a patient-specific IED spike template to determine the most relevant component. Ultimately, the time series of this component was convolved with the HRF, resampled to the frequency of the fMRI recording, and used in the GLM analysis. To evaluate this method quantitatively, we calculated the distance from the dipole locations in EEG source localization to the BOLD cluster and compared the results to those obtained from the conventional methods.

## Materials and Methods

### Subjects

30 patients from Epilepsy Center, Pars Hospital, Tehran, Iran, who were surgery candidates with focal or generalized epilepsy and also would show at least 10 distinct IEDs during the MRI scanning were included in the study. The ethical approval was obtained from the ethics committee of the Iran University of Medical Sciences, Tehran, Iran, and all patients provided written informed consent.

### Long-Term Monitoring (LTM)

All the patients underwent a long-term 64-channel EEG with 500 Hz sampling rates and following the 10–20 standard for electrode placement as a preoperative evaluation at the Epilepsy Center, Pars Hospital, Tehran, Iran. Besides, all the other available information such as the comprehensive clinical record, full neurological examination, neuropsychological assessment, structural MRI, and other non-invasive investigations like ictal SPECT and PET were reviewed to help the localization of irritative zone (IZ) and Seizure Onset Zone (SOZ) through the preoperative evaluation.

### EEG-fMRI Acquisition

Simultaneous recording of EEG-fMRI was performed from May 2017 to June 2018 at the National Brain Mapping Laboratory (NBML), Tehran, Iran, in the form of 20-mins sessions with eyes closed. The MRI scanner was the 3 T Siemens Prisma, and the EEG amplifier was a 64-channel BrainAmp MRI-compatible system from Brain Products with 5 kHz sampling rates. The EEG internal clock was synchronized with the MRI clock and the EEG electrodes followed the 10–20 placement system with the reference of Cz. Besides, the ECG signal was recorded using a bipolar lead (10, 28), and a 10-min EEG recording was acquired with eyes closed outside the scanner immediately before the EEG-fMRI session (28). EEG electrodes were equipped with an additional 5 kΩ terminal resistance, and impedances were kept as low as possible to improve the quality of the recording.

For the MRI scanning, a T1 MPRAGE anatomic sequence was first scanned [1 mm slices, 256 × 256 matrices, echo time (TE) = 3.74 ms, repetition time (TR) = 1,810 ms, flip angle = 30°] to use in registering functional images. Functional data was obtained in 20-min runs with patients at rest, using a T2^*^-weighted gradient-echo (GRE) imaging sequence (234 × 234 matrix, 40 slices, 3 × 3 × 3 mm, TE = 26 ms, TR = 2,500 ms, flip angle = 60°) (29). To minimize the movement of the patient's head and provide comfort, a pillow filled with foam microspheres was used inside the scanner.

### EEG Signal Processing—Long Term Monitoring

The EEG signals were preprocessed using the EEGLAB toolbox (https://sccn.ucsd.edu/eeglab/). First, the sampling rate of the signal was reduced to 250 Hz, and a Butterworth high-pass filter at 1 Hz was used to suppress the low-frequency components (30–32). Then, all the channels were reviewed, and those with a standard deviation greater than ±3.1 from the mean standard deviation (across all channels) were excluded as the abnormal channels. For eliminating the power-line noise at 50 Hz, the Clean Line algorithm was used (29). The advantage of this algorithm over the notch filter is that it adaptively estimates and removes sinusoidal artifacts without creating band-holes in the EEG power spectrum (29, 33).

**Figure 1:**
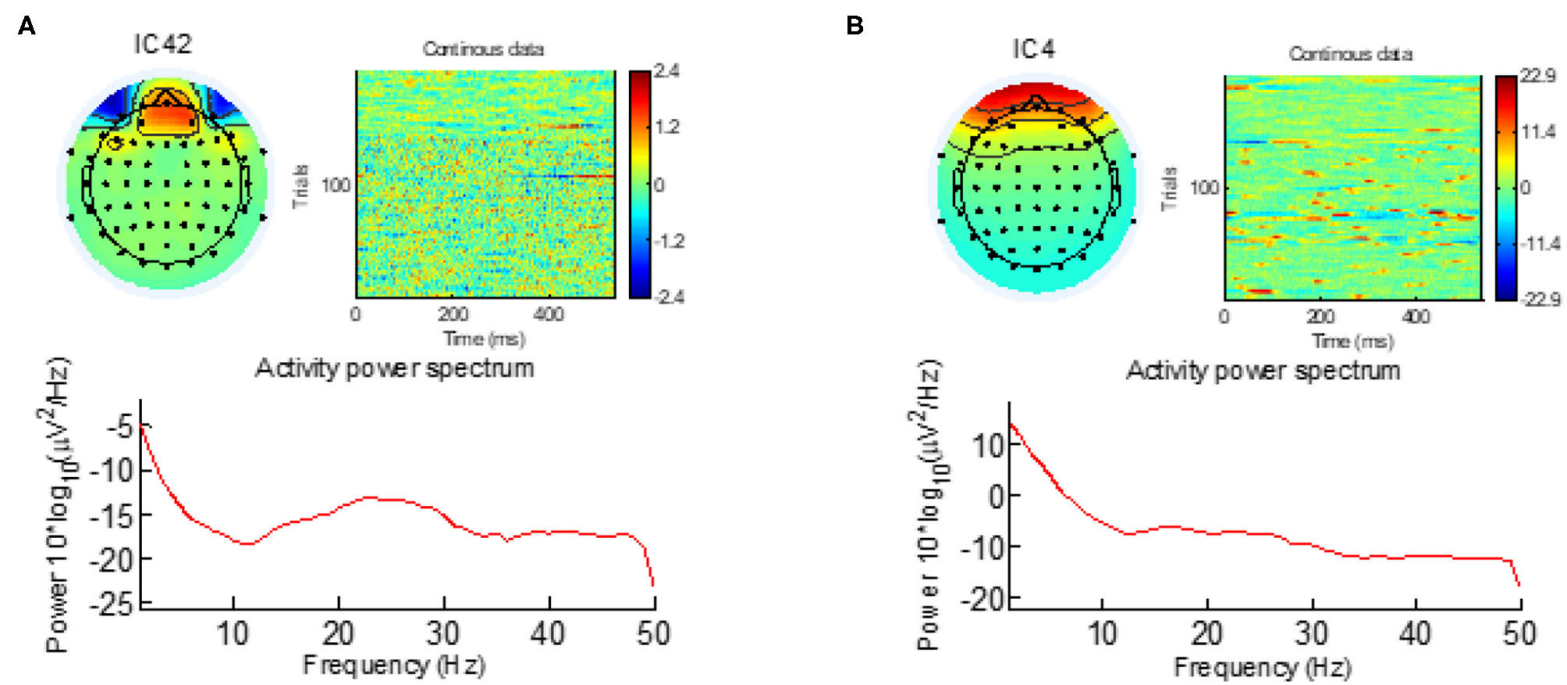
The eye-blinking and eye-movement artifacts are visible both in the scalp map and in the activity power spectrum. **(A)** Eye-blinking artifact. **(B)** Eye-movement artifact.

Next, the ICA algorithm was applied on the EEG signal and the irrelevant components corresponding to eye blink, eye movement, cardiac pulsatile, muscular tension, swallowing, or machine vibration were visually identified using the component's scalp map, spectral power activity, and spectral power distribution. Figure 1 shows typical samples of two such components identified as artifacts. After identifying all the artifact components, the data were re-composed without them.

The resultant cleaned signals were evaluated by a trained expert, and the IEDs were marked for the main analysis. Then, the IEDs were averaged to build patient-specific and morphology-specific IED templates. After band-pass filtering (34) (1–30 Hz) and epoching the spikes with a fixed length of 0.3 s and step size of 1 sample (2.5 ms) to include the negative peak and slow-wave, the template of the spike was set for each subject by hand-selecting and averaging 10–20 spikes from the marked signal by an experienced electroencephalographer. New spikes would then be detected and added to the initial template (29). In case a patient had more than one type of spike, this process is separately done for each different type (35). The match between the template, x, and each 300 ms of candidate component at the times of the IED, y, was defined as the magnitude of the sample correlation, |*r*_x*y*_|, presented in Eq. 1.


(1) $$\begin{array}{l} r_{xy}=\;\frac{\sum_{i=1}^{n}\left(x_{i}-\bar{x}\right)\left(y_{i}-\bar{y}\right)}{\sqrt{\sum_{i=1}^{n}{\left(x_{i}-\bar{x}\right)}^{2}\sum_{i=1}^{n}{\left(y_{i}-\bar{y}\right)}^{2}}} \end{array}$$

To add to the initial, hand-selected template, a first pass over the data was performed at a high threshold (*rxy* = 0.96–0.98 depending on the subject) (36).

### EEG Signal Processing—Simultaneous EEG/fMRI

**Figure 2:**
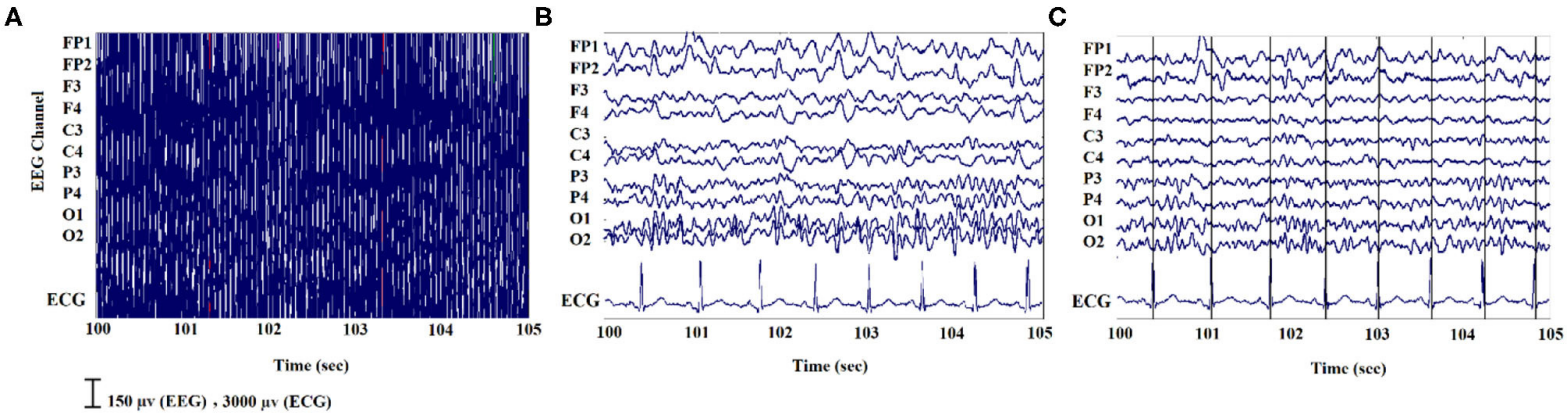
An illustration of EEG signals recorded inside the scanner: **(A)** noisy signal; **(B)** after removing the MR gradient artifact; **(C)** after eliminating the BCG artifacts.

For the EEG signals recorded inside the scanner, the MR gradient switching artifact was eliminated using the fMRIb algorithm (https://fsl.fmrib.ox.ac.uk/eeglab/fmribplugin/) which first increases the sampling rate to 20 kHz, and then applies a low-pass filter at 60 Hz (37). Also, the ballistocardiogram (BCG) artifact that occurs because of the movements of the electrodes associated with cardiac pulsations, was detected and removed with the fMRIb toolbox using the heartbeat information from the extra ECG electrode during EEG-fMRI recording. A sample of EEG signals recorded inside the MR scanner before and after removing the MR gradient and BCG artifacts are shown in Figure 2.

### Template Component Cross-Correlation (TCCC) Method

**Figure 3:**
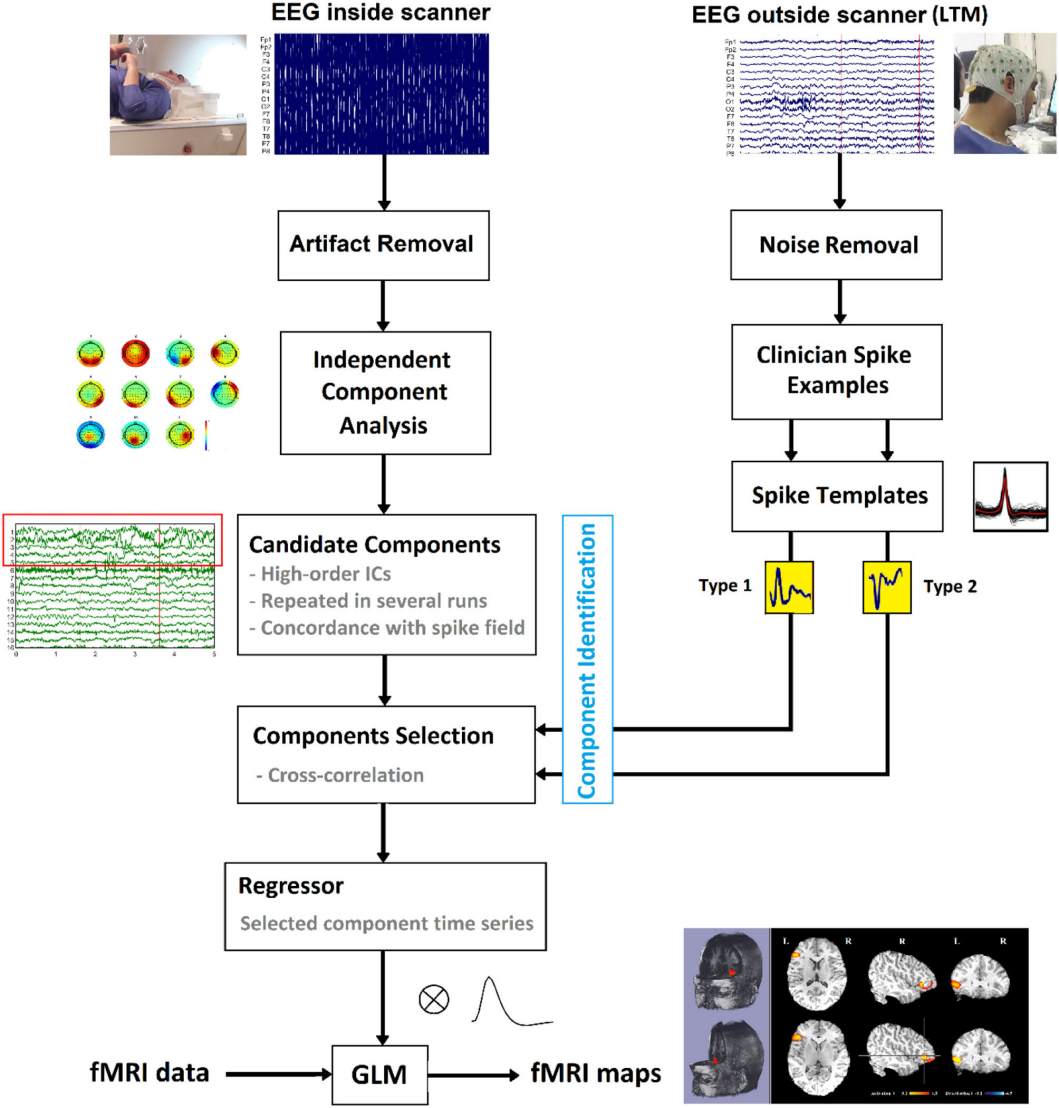
Schematic of the suggested method to localize the epileptic foci using simultaneous EEG-fMRI.

The general pipeline of the proposed method is summarized in Figure 3. First, the EEG signal recorded outside the scanner was preprocessed, and the individual patient-specific IED templates were extracted for each subject. Besides, the EEG signal recorded inside the scanner underwent ICA analysis and was decomposed to its independent components after artifact removal for selecting a set of candidate components representing actual generators of epileptic activity. Different ICA algorithm parameters can lead to various components but if the candidate components are reliable sources, they should be robust to variations in the ICA decomposition process. Therefore, the ICA algorithm was applied 10 times using different arbitrary (random) initialization weights, and the initial candidates selected based on being those seen most often in the 10 repetitions (38). From these, the three components with the highest average λ (weight of extracted independent components) across all 10 iterations were selected as final candidates.

**Figure 4:**
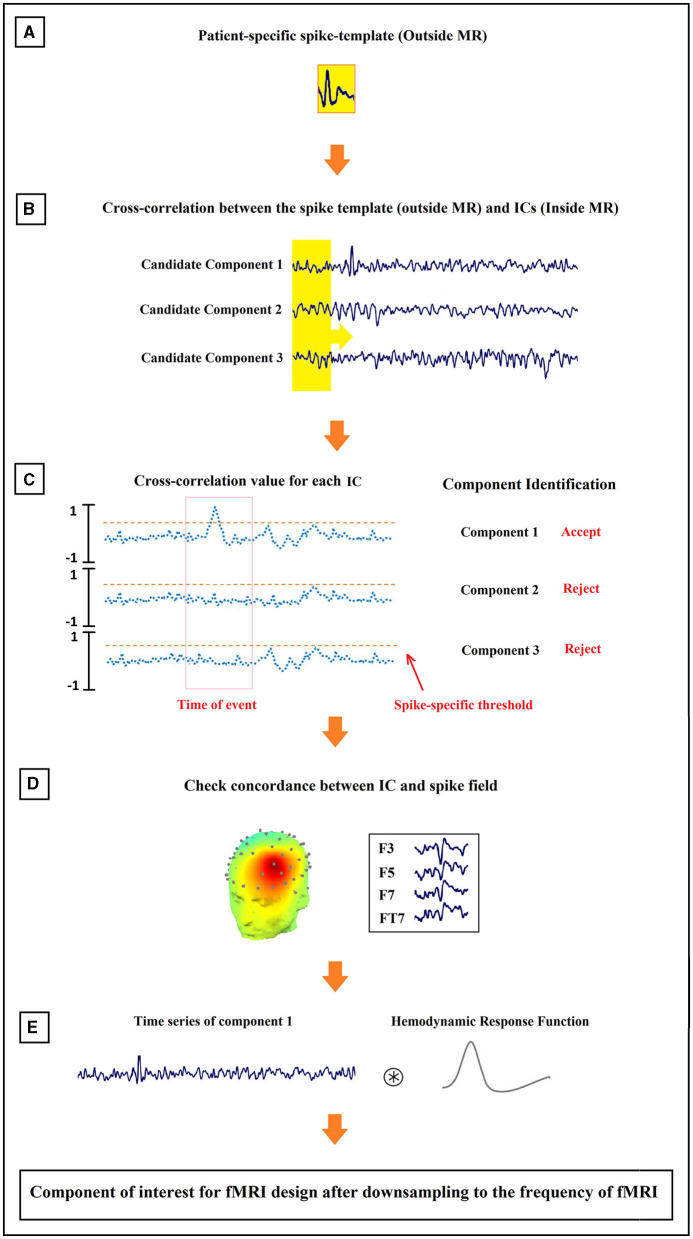
Schematic illustration of the method proposed for the identification of epilepsy-related components. The order of the process from **(A–E)** is shown in the figure.

The set of final candidate components of each patient underwent the analysis of cross-correlation with their specific IED templates, built earlier from Long Term Monitoring (LTM) data (Figure 4A). The process employed a sliding window of width 0.3 s and step size of 1 sample (the yellow box with the arrow in Figure 4B). EEG inside scanner was marked by an experienced electroencephalographer and the marked times were used for the cross-correlation. Components that did not have cross-correlation with the templates at the times of the IED events of at least 0.85 were rejected (Figure 4C). Also rejected were the candidates judged to be discordant with the observed IEDs in the EEG (more than 50 mm away) (Figure 4D).

The time course of each remaining component was assumed to be the temporal activity of an epileptic source. So, they were convolved with the canonical HRF (Figure 4E), resampled to the frequency of the fMRI recording (TR = 2.5 s), and used as the predicted model in the GLM analysis. For multiple spikes, the regressors of the different types were entered simultaneously into a single first-order analysis.

### fMRI Analysis

In the conventional analysis, the EEG signals recorded simultaneously with fMRI are reviewed and marked for determining IEDs as zero-duration events, and the resultant time series is convolved with a standard HRF for use in the GLM analysis as the regressor of epileptic activity. However, in our Template Component Cross-Correlation (TCCC) method, the time series of the epileptic-related components are convolved with four HRFs, peaking at 3, 5, 7, and 9 s (12).

The fMRI dataset was preprocessed and analyzed using FSL (FMRI Expert Analysis Tool, Version 6.0.1, FMRIB's Software Library, http://www.fmrib.ox.ac.uk/fsl). Motion correction was done via a 6-parameter rigid-body transformation, and the dataset was spatially smoothed via 6-mm full width at half-maximum. Also, an autoregressive model of order one was used to correct the temporal autocorrelations (39), a third-order polynomial was used to model low-frequency drifts and applied as high-pass temporal filtering. For each fMRI dataset, all of the models built from each of its IED components were included in the same GLM, thus total variance was partitioned amongst the inputs, effectively treating the others as confounds.

All regressors were included in the same GLM in the fMRI analysis (fMRIstat). For each event type, a statistic z-map was created for each regressor using the other regressors as confounds. A combined z-map was created by taking, at each voxel, the maximum z-value from the four z-maps based on four HRFs. The single combined t-map was used for the subsequent analysis. For the second-level analysis, each cluster with at least five contiguous voxels having a *z-*value >3.1, corresponding to a *p* value smaller than 0.05 was selected as the significant result. This included correction for multiple comparisons, accounting for the number of voxels and the 4 HRFs. The final statistical maps were then registered to and overlaid on the patient-specific structural MRI. In the *z*-maps, a yellow-red scale corresponds to positive BOLD responses (activation) and a blue-white scale to negative responses (deactivation). Responses outside the brain were excluded and BOLD responses in the ventricles were excluded using a mask, as they are often interpreted as artifactual findings.

### Concordance Between IED Location and Maximal BOLD Response

For evaluating the results of the analysis, the spatial concordance between the BOLD response and the IED field was assessed. First, the locations of the single voxel with maximal z-score of the maximum BOLD cluster and the extracted dipole from ICA algorithm were determined. Next, the distance between the locations was measured and classified into three levels of spatial concordance: (i) concordant (C) for the distance <25 mm; (ii) partially concordant (PC) for the distance between 25 and 50 mm; and (iii) discordant (D) for the distance more than 50 mm (36, 40). To evaluate the distance between dipole and maximum BOLD, the spherical head model has been co-registered to the MNI brain. Spherical dipoles coordinates are also converted to MNI. The fMRI data is also co-registred and normalized into a MNI atlase.

### Contribution of TCCC Method

The evaluation of IED sources and seizure onset zone is usually involved in the standard clinical practice for the planning of surgical resection in epilepsy. However, the use of simultaneous EEG-fMRI is not currently part of such standard practice. If it presents meaningful information for more precise localization of the IED sources, it may become a helpful part of the standard clinical practice for presurgical evaluation. Therefore, we have included the evaluation of our final BOLD results for each type of IED in terms of their potential contribution. We defined the BOLD results as contributory if: (i) the BOLD response detected the IED generation field with higher precision and accuracy than EEG source localization, and (ii) the maximum BOLD response was in deep brain structures compared to the surface location of the recorded IED.

If there was no concordance between the BOLD response and the IED field or the results had no new information beyond that provided by the EEG signals, it was not labeled as contributory. Besides, another comparison was made between the the lesional findings of the structural MRI and our BOLD response to ensure precision that possible contributory effects of an MRI lesion on the BOLD response were also taken into account.

## Results

From the total of 34 patients who were recruited for the study and underwent EEG-fMRI, four were not included because of having no clear IEDs during the EEG-fMRI session or significant movement artifact during recording. Table 1 shows the summarized clinical details for the remaining 30 patients who were 16 females and 14 males (15–48 years with the mean of 27.3 years) and a seizure onset age of 1–22 years (mean of 10.6 years). 28 of the patients had focal epilepsy which 23 of them were unifocal, four were bifocal, and one was multifocal. The other two were generalized epilepsy patients which one of them had continuous spikes and waves during slow sleep (CSWS) and the other one had West's Syndrome. These classifications were done by two expert neurologists before EEG-fMRI recording, based on structural MRI, EEG signals, and clinical records of patients. 6 of the patients in the focal group had lesions on their MRI scans. For each patient, the number of spikes in the routine EEG and during EEG-fMRI recording is listed in the last columns of Table 1.

**Table 1:** Patients clinical information.

**No**.	**Sex/Age**	**Age of onset**	**Type of epilepsy**	**AED**	**Ictal EEG Onset**	**Interictal EEG**	**No. of spikes in routine EEG**	**No. of spikes during fMRI**
1	F/15	1	TLE, R	VPA, LEV	Tempo. R	Tempo. R	25	17
2	M/14	18	FLE, L	LCM, LTG	Fronto-cent. Bil	Front. L	38	22, 18
3	M/19	10	FLE, R	LCM, OXC, LEV	Front. R	Front. R	49	14, 18, 16
4	F/23	8	PLE, L	OXC, LEV	Pariet. L	Pariet. L	46	38
5	M/34	7	TLE, L	LEV, VPA	Tempo. L	Fronto-tempo. L	33	13, 15
6	F/28	3	IE	OXC, LTG, PT	Bil Gen.	Bil Gen.	36	21
7	F/48	8	FLE, L	LEV	Front. L	Fronto-cent. L	37	18, 9, 11
8	M/32	12	TLE, R	LTG, LCM	Tempo. R	Tempo. R	31	28
9	M/17	1	FLE, R	OXC, VPA, TPM	Front. R/L	Front. L	26	19
10	F/29	20	Unclear	OXC	Hemisphere L	Fronto-temp. L	33	27
11	M/28	16	OLE, R	LTG, OXC	Occip. R	Occip. R	61	14, 18, 25
12	F/18	3	SE	OXC, LTG	Bil. Front.	Bil. Front.	29	13,7
13	M/36	7	Multifocal: P/T	VPA, TPM, LEV	L Pariet./Post Tempo.	L Pariet./Post Tempo.	25	22
14	M/24	15	FLE, R	LTG	Frontopolar R	Fronto-cent. R	34	13, 8, 9
15	F/33	9	TLE, R	CMC, OXC	Tempo. R	Tempo. R	35	38
16	F/40	16	PLE, L	TPM, VPA,	Parieto-occip. L	Pariet. L	26	32
17	F/25	13	Unclear	LEV, OXC	Fronto-cent. Bil	Front. R	38	44
18	F/21	10	FLE, L	LTG, CLO	Fronto-tempo. L	Fronto-tempo. L	41	21, 12, 11
19	M/28	17	PLE, R	CMC, LTG	Pariet. R	Pariet. R	28	35
20	F/19	1	TLE, R	LEV, ESM, ZSM	Tempo. pole	Tempo. R	26	29
21	M/41	16	TLE, R	VPA, CMC	Tempo. R	Tempo. R	28	21
22	M/24	17	FLE, L	LTG	Fronto-tempo. L	Fronto-tempo. and Tempo. L	33	26, 17
23	F/16	3	PLE, R	OXC	Pariet/Occip R	Pariet R	27	10, 19
24	M/18	8	FLE, L	LEV, VPA	Fronto-tempo. L	Front. L	18	13
25	F/28	21	TLE, L	LEV	Tempo. L	Tempo./ Pariet. L	45	28, 16
26	F/32	22	FLE, Bil	LEV, OXC, LCM	Front. Bil.	Front. L>R	17	19
27	M/36	7	TLE, R	OXC	Fronto-tempo. R	Tempo. R	29	32
28	F/26	15	FLE, L	VPA	Front. L	Tempo./ Front. L	25	19
29	F/38	14	TLE, R	VPA, LTG	Tempo. Bil	Tempo. R	37	24, 19
30	M/20	2	OLE, R	TPM, OXC	Pariet./Occip. R	Occip. R	47	43

*AED, antiepileptic drug; Bil, bilateral; CMC, carbamazepine; CLO, clobazam; ESM, ethosuximide; FLE, frontal lobe epilepsy; Gen, generalized; IE, idiopathic epilepsy; L, left; LCM, lacosamide; LEV, levetiracetam; LTG, lamotrigine; OLE, occipital lobe epilepsy; OXC, oxcarbazepine; Pariet., Parietal; PT, phenytoin; PLE, parietal lobe epilepsy; R, right; SE, symptomatic epilepsy; P, parietal; TLE, temporal lobe epilepsy; VPA, valproate; TPM, topiramate; ZSM: zonisamide (29)*.

**Figure 5:**
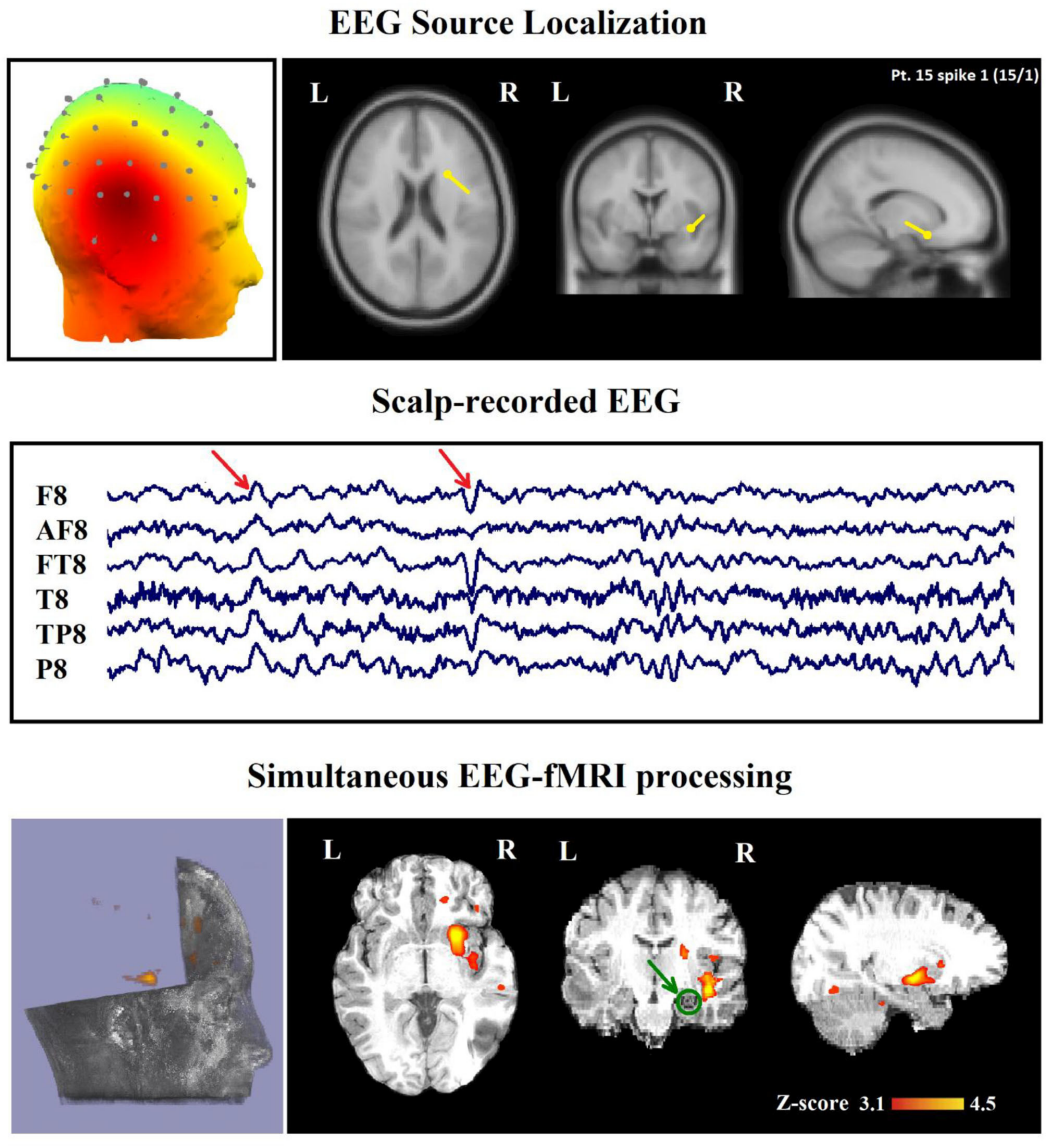
(Patient 15, spike 1) Marked events are F8, FT8 spikes, and the TCCC-related BOLD response shows a neocortical activation in the right head and the superior temporal gyrus. This response is considered concordant with the spike field but not contributory. Based on the mesial temporal sclerosis (MTS), the patient has independent validation information, but the response is invalidated. The green circle shows the MTS area. Top, the component identified on scalp EEG located in the right temporal lobe (left) and the dipole localization of the identified generator in deep brain structures (right) based on analysis of EEG inside the scanner. Middle, scalp recorded EEG. Bottom, Localization of the generator applying simultaneous analysis of EEG-fMRI. The active area is marked with a yellow-red color.

**Figure 6:**
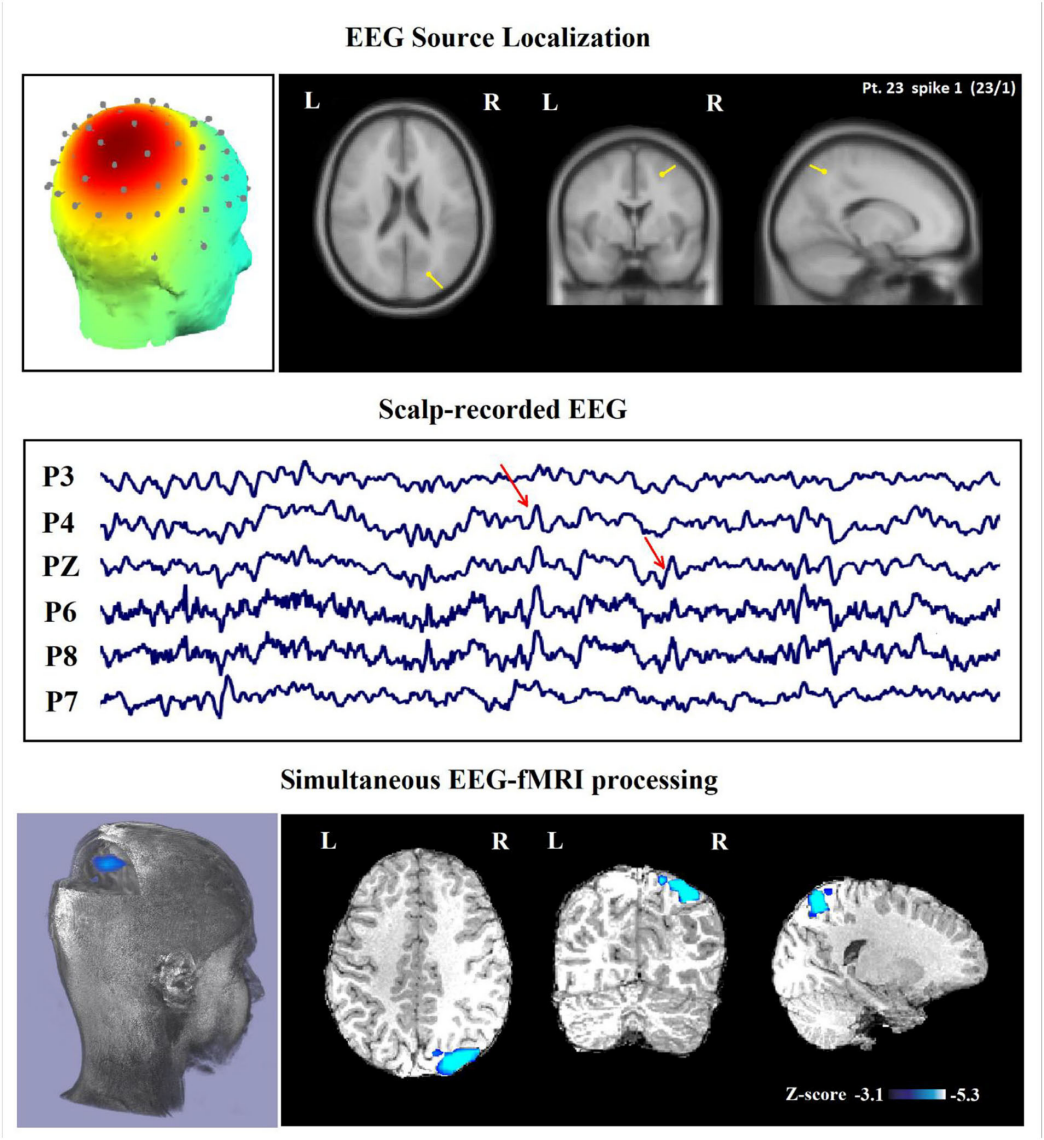
(Patient 23—spike 1) marked events are parietal spikes and wave complexes (referential montage). The TCCC-related BOLD response shows deactivation in the posterior part of the right superior parietal lobule. This BOLD response is considered concordant with the spike field and contributed to a better localization of the epileptic foci compared with the scalp EEG. Top, the component identified on scalp EEG located in the right middle parietal lobe (left) and the dipole localization of the identified generator in brain structures (right) based on analysis of EEG inside the scanner. Middle, scalp recorded EEG. Bottom, Localization of the generator applying simultaneous analysis of EEG-fMRI. The deactivation is marked with a blue-white color.

**Figure 7:**
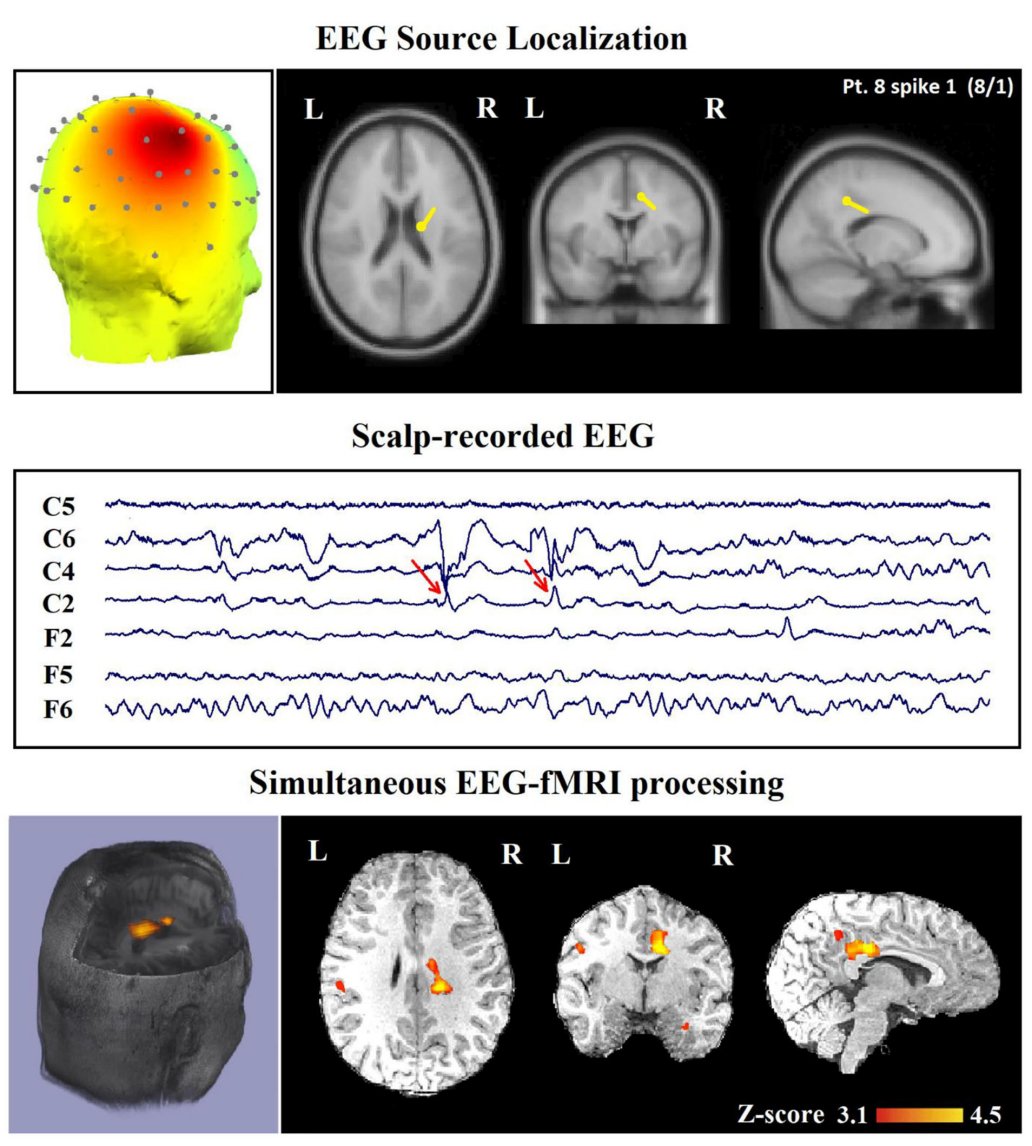
(Patient 8—spike 1) marked events are C4–C6 spikes (referential montage), and TCCC-related BOLD response shows a focal activation in the right head. This response is considered concordant with the spike field and contributory, because it shows the involvement of a deep brain structure, in the epileptic focus, which is not visible on the scalp EEG, based on anatomo-electroclinical correlations. The focus was identified in the cerebral medulla (with matter). Top, the component identified on scalp EEG located in the right temporal lobe and the dipole localization of the identified generator in deep brain structures. The active area is marked with a yellow-red color. Middle, scalp recorded EEG. Bottom, Localization of the generator applying simultaneous analysis of EEG-fMRI.

**Figure 8:**
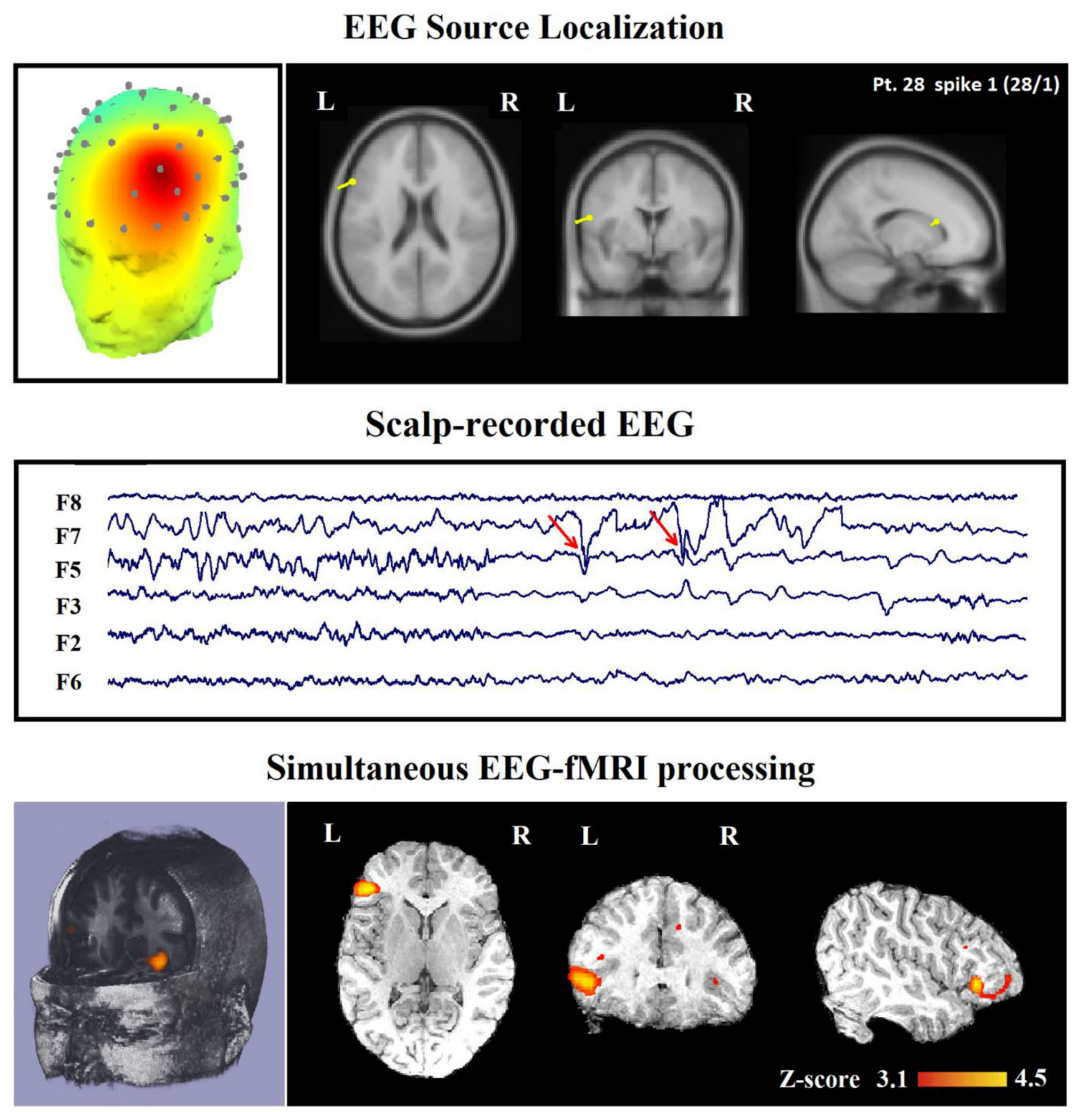
(Patient 28—spike 1) marked events are F3, F5, and F7 spikes (referential montage) and the TCCC-related BOLD response demonstrates a neocortical activation in the inferior frontal gyrus. This response is considered concordant with the spike field but not contributory because it does not add any new information to the scalp EEG. Top, the component identified on scalp EEG located in the left frontotemporal lobe (left) and the dipole localization of the identified generator in deep brain structures (right). Middle, scalp recorded EEG. Bottom, Localization of the generator applying simultaneous analysis of EEG-fMRI. The active area is marked with a yellow-red color.

**Figure 9:**
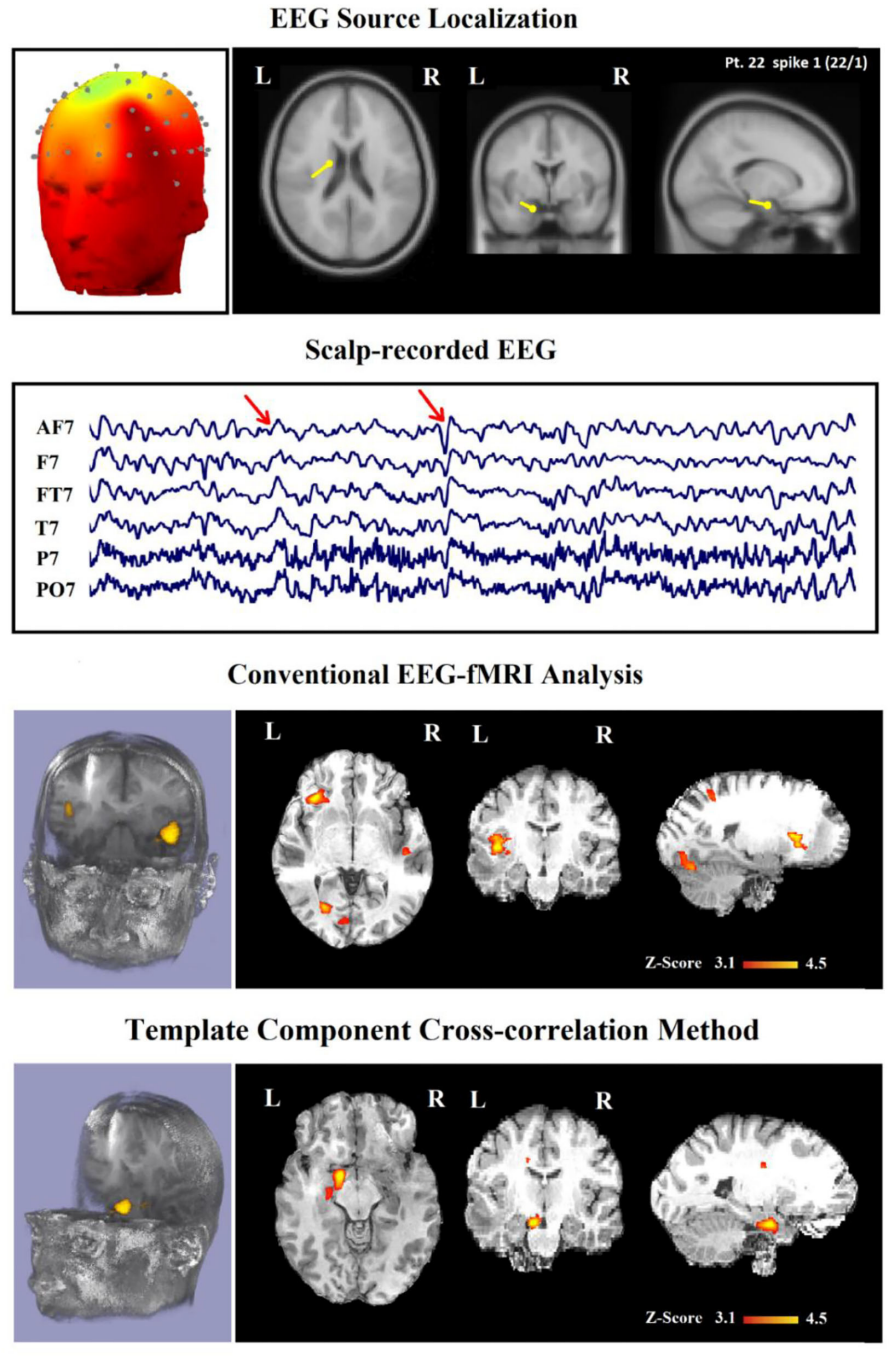
(Patient 22—spike 1) marked events are AF7, T7, and FT7 spikes (referential montage) and the TCCC-related BOLD response demonstrates a neocortical activation in the caudate nucleus and lentiform nucleus for the TCCC method, this response is considered both concordant with the spike field and contributory duo to it leads to better localization of the epileptic focus compared with the scalp EEG because it adds new information to the scalp EEG, while is considered partially concordance and not contributory for the conventional method. These foci are scattered in three areas. Top, the component identified on scalp EEG located in the left frontotemporal lobe and the dipole localization of the identified generator in deep brain structures. The active area is marked with a yellow-red color. Second row, scalp recorded EEG. Third row, localization of the generator applying simultaneous analysis of EEG-fMRI by the conventional method. Forth row, localization of the generator applying simultaneous analysis of EEG-fMRI by the TCCC.

From the total number of patients, seven had two types of IED, five had three types of spikes, and the rest had one type of spike. We generated one study for each type of spike for the analysis of the TCCC method. Therefore, a total of 792 IEDs from 47 IED-studies underwent EEG-fMRI analysis. Two of the patients with multiple IED types showed no BOLD response. In all of the other 45 studies, at least one BOLD response was observed; 19 had spike-associated activation only (Figures 5, **7**, **8**), 9 had spike-associated deactivation only (Figure 6), and 17 had spike-associated activation and deactivation.

### Concordance Between TCCC-Related BOLD Changes and Identified Component-Related Dipole

After calculating the distance between the center of gravity for maximum BOLD clusters and center of identified component-related dipole for all 45 IED-analyses, the result of statistical analysis showed that the distances between spike field and BOLD cluster for discordant (D) (>50 mm), partially concordant (PC) (25–50 mm), and concordant (C) (<25 mm) groups were significantly distinct from each other (*p* < 0.0001). Overall, 35 types of IED were concordant (13.83 ± 5.37 mm), 9 types of IED were partially concordant (32.44 ± 7.24 mm), and 1 was discordant to the relevant BOLD cluster (*p* < 0.0001).

In 29 patients out of 30 (97%), a minimum of one concordant TCCC-related BOLD response (35 analyses) with the identified component location was found (Figures 5, 6). These concordant responses were generalized in 2 patients and focal in 27 ones who had focal discharges. Less significant responses were found in the rostral anterior cingulate gyrus, hypothalamus or posterolateral and occipital areas.

The highest activations and deactivations were found in four cases who had bilateral diffuse discharges at the posterior cingulate or the parietal areas (default mode regions) and the anterior cingulate or hypothalamus, respectively. Patient 3 had right FLE symptomatic of a small area of focal cortical dysplasia. The BOLD response to the identified components was spatially concordant with the lesion.

Only one of all the 30 patients with one IED-study (patient 10) (3%), who was not a candidate for surgical resection because of poor clinical seizure focus, had a partially concordant BOLD response with the dipole. This patient with left frontotemporal discharge did not show any significant BOLD changes at the region of the identified component. However, a BOLD response was found with a maximum z-score in the contralateral parietal region.

### Contribution of TCCC Method to Defining SOZ

In 24 patients (80%), a minimum of one contributory significant BOLD change (29 analyses) was found (Figures 6, 7). In 19 of them, the TCCC-related BOLD change was more capable in comparison with EEG alone to identify the cortical region that generates the spike (Figure 6): frontal lobe in 8 (patients 3, 5, 7, 9, 12, 14, 18, and 28), temporal lobe in 4 (patients 1, 20, 25, and 29), parietal lobe in 5 (patients 13, 16, 17, 19, and 23), and occipital lobe in 2 (patient 11 and 30). In the remaining 5 subjects, the maximum TCCC-related BOLD changes were found in deep brain structures, which are most probably involved in the epileptogenic zone (Figure 7): basal ganglia and amygdala in 2 (patient 22, 27), and heterotopic tissue in three with nodular heterotopias (patients 2, 8, and 21).

In 16 of the 45 IED studies, the most clinically relevant BOLD response was not contributory. In 9 of them, the TCCC-related BOLD change was partially concordant with the identified component location. In 1 IED study, it was discordant, in 5 IED studies, the BOLD response did not provide any new information in comparison with EEG alone (Figure 8), and in the last one (patient 15, spike 1), it was invalidated (Figure 5).

For evaluating the TCCC method in patients with an epileptic lesion localized by structural MRI, we drew our attention to six of these patients with focal epilepsy, and it revealed concordant, contributory, and validated BOLD response in five of them. The only subject (patient 15) who showed invalidated response had a right frontal MTS but only a neocortical right temporal activation (Figure 5).

In comparison between the TCCC method and conventional EEG-fMRI analysis, the localization of the TCCC-identified component was concordant with the epileptogenic area of conventional analysis for 35 out of 45 IED studies (77%). This clearly shows the accuracy of the TCCC method for detecting the epileptic generators by studying the component of interest, confirming the detected generator's temporal behavior.

### Comparison With Conventional Spike-Related Analysis

**Figure 10:**
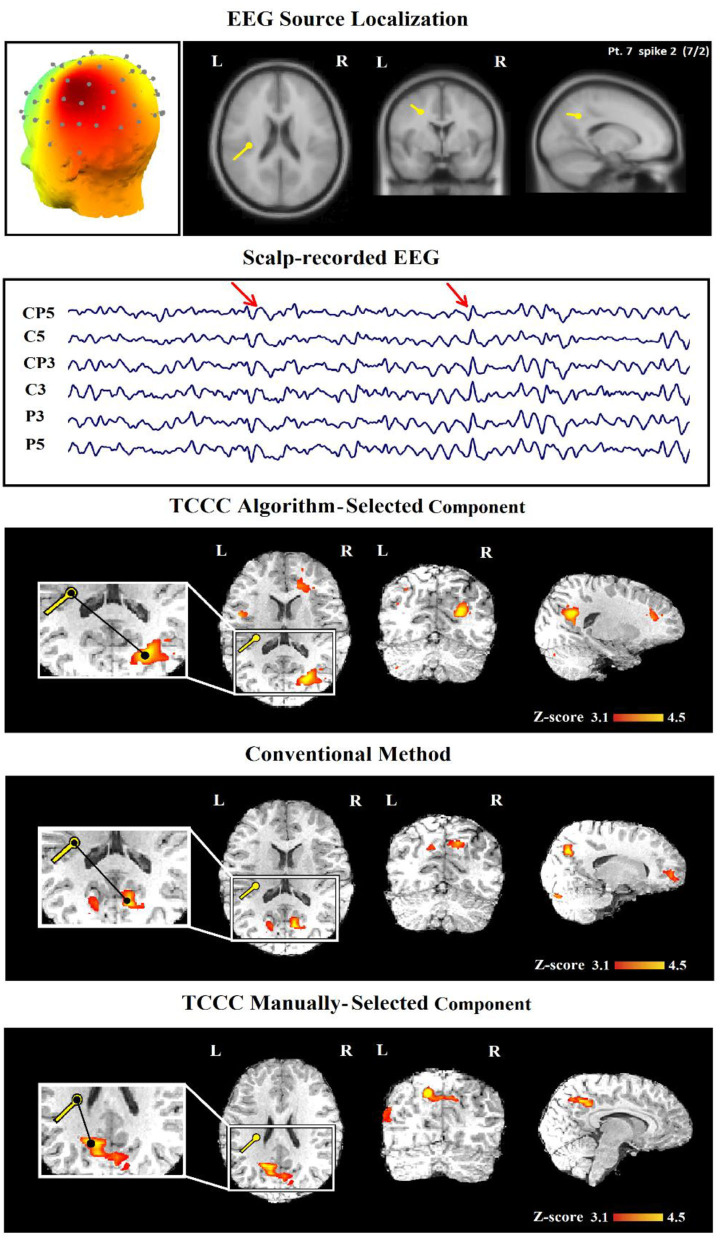
(Patient 22—spike 2) An illustration of a sample result for conventional and TCCC methods both manually and automatically. First row, the identified component and its dipole localization in deep brain structures. Second row, scalp recorded EEG. Third row, TCCC-related BOLD response using auto-identified component (Discordant). The active area is marked with a yellow-red color. Forth row, IED-related BOLD response (conventional method). Fifth row, TCCC-related BOLD response using manually-selected component (Concordance).

In Table 2, we present a comparison between the spatial distribution of spike-related and TCCC-related BOLD responses. The spatial concordance of the BOLD responses of the conventional method with the electroclinical evaluation was found in 14 of the 30 patients. All of these patients also showed concordant TCCC-related BOLD responses with the results of conventional analysis and validated by clinical records of the patient and the site of the surgical resection. The results of the TCCC method were better than the conventional analysis in 7 patients for determining the cortical spike generator region. No clear IEDs were found during the EEG-fMRI recording in one of them, the significant BOLD changes was not concordant with the spike field in the remaining 6 patients. Unlike other medical information, the results of the conventional analysis in these 6 patients showed no clear foci or multiple potential distinct foci. However, the BOLD responses of the TCCC method revealed a circumscribed foci within the expected region (Figure 9). In 3 of the 45 IED studies, the TCCC method was weaker than the conventional analysis (Figure 10)

**Table 2:** Comparison of localization between conventional method and TCCC-method.

**SOZ (No.)**	**Epileptic types**	**TCCC-method**	**Conventional method**
Generalized (2)	CSWS (1)	1	–
	Syndrome (1)	1	1
Unifocal (23)	Non-lesional (17)	10	8
	Lesional (6)	5	3
Bifocal (4)		3	2
Multifocal (1)		1	–
Total	SOZ-based (30)	21, 70%	14, 46%
	Spike-type analysis (45)	29, 64%	21, 46%

The concordance of the results of the TCCC method and the conventional analysis is shown in Table 3. The most obvious information that can be extracted from the table are: (1) a satisfactory agreement between the results of the TCCC method and the conventional method in 32 IED studies; (2) a higher maximum z-score in 28 patients and greater extent of activation in 22 of patients using TCCC method; and (3) different activation regions in three patients with deep located epileptic foci, and no apparent agreement in one patient with deep epileptic foci and also no noticeable activation using the conventional method. This suggests that the conventional method is less effective when the epileptic generator is located remote from the scalp.

**Table 3:** Comparison of concordance between conventional EEG-fMRI analysis and TCCC method in relation to BOLD response and EEG data of 47 IED studies.

**Pt/IED type**	**IEDs Location**	**Template Component Cross-correlation EEG-fMRI**						**Conventional EEG-fMRI**						**Agreement between TCCC and conventional methods**
		**Dis.^a^** **(mm)**	**Conc.^b^**	**Max Z score for relevant cluster**	**Max BOLD volume** **(cm^3^)**	**BOLD in other location(s)**	**Contribution**	**Dis.^a^** **(mm)**	**Conc.^c^**	**Max Z score for relevant cluster**	**Max BOLD volume (cm^3^)**	**BOLD in other location(s)**	**Contribution**	**Concordance^d^/Dis. (mm)**
1/1	T R	21.33	C	3.84	1.22	L lateral T	Yes	19.52	C	3.63	0.98	L mesial T	Yes	Yes/5.23
2/1	FC bil	17.80	C	4.10	3.51	–	Yes	20.14	C	4.18	2.94	L lateral T	Yes	Yes/6.01
2/2	FC L	14.35	C	2.22	1.12	–	Yes	16.39	C	6.15	1.04	–	No	Yes/4.28
3/1	FT R	19.32	C	3.17	1.94	L mesial P	Yes	28.45	PC	4.52	3.12	–	No	Yes/11.49
3/2	F R	42.15	PC	3.45	2.51	–	No	51.64	D	3.66	2.43	L middle T gyrus	No	Yes/18.54
3/3	Bif	15.25	C	4.07	3.73	–	Yes	18.25	C	4.12	4.26	–	Yes	Yes/7.64
4/1	P L	11.61	C	5.26	4.12	–	No	12.35	C	4.53	1.15	–	Yes	Yes/9.23
5/1	T L	36.42	PC	4.01	0.75	LP–O	No	30.25	–	–	–	–	–	–
5/2	FL	9.34	C	6.95	0.86	–	Yes	14.28	C	5.42	2.51	L anterior P	Yes	Yes/15.81
6/1	Bil generalized	17.55	C	3.87	10.23	–	No	18.41	C	3.54	8.79	–	No	Yes/2.18
7/1	F L	10.48	C	5.16	1.64	L anterior P	Yes	13.25	C	4.56	1.88	Bil F–P	Yes	Yes/22.37
7/2	C L	50.24	D	4.46	2.65	R middle F	No	43.25	PC	3.56	1.02		No	No/12.65
7/3	T R	26.01	PC	5.13	2.41	–	No	18.98	C	16.14	0.89	mesial T–P	Yes	Yes/17.24
8/1	T R	7.61	C	4.38	1.32	L lateral T	Yes	32.27	PC	4.85	5.27	L anterior F	No	No/26.55
9/1	F R/L	8.04	C	6.23	3.18	–	Yes	13.52	C	5.93	3.26	L posterolateral F	Yes	Yes/6.24
10/1	L hemisphere	38.25	PC	3.87	5.44	L P	No	55.14	D	3.36	5.36	–	No	Yes/15.67
11/1	O R	14.32	C	4.29	1.01	L anterior P	Yes	12.62	C	4.11	0.98	L orbito–F	Yes	Yes/8.45
11/2	PO R	–	–	–	–	–	–	51.56	D	2.14	3.14	–	No	–
11/3	P R	23.25	C	6.52	8.42	–	Yes	19.25	C	5.34	2.64	–	Yes	Yes/5.06
12/1	Bifrontal	37.52	PC	5.13	2.80	–	No	35.14	PC	5.09	3.02	–	No	Yes/4.34
12/2	F L	13.46	C	4.58	0.98	–	Yes	13.46	C	6.18	1.06	–	Yes	Yes/0.09
13/1	P L/Post T	16.18	C	3.28	1.62	–	Yes	28.62	PC	4.16	1.15	–	No	No/27.35
14/1	Frontopolar R	22.57	C	4.49	3.46	–	Yes	21.78	C	4.28	3.61	–	Yes	Yes/2.42
14/2	FT R	30.24	PC	4.68	2.16	–	No	32.47	PC	3.74	2.13	–	No	Yes/5.67
14/3	FT L	21.85	C	5.85	0.61	–	Yes	26.85	PC	4.88	0.24	L post T–O	No	No/29.13
15/1	T R	5.34	C	4.57	1.16	R anterior F	No	13.25	C	6.11	0.76	–	No	Yes/10.16
16/1	PO L	18.62	C	4.36	2.53	–	Yes	19.77	C	4.28	2.84	–	Yes	Yes/3.49
17/1	FC bil	9.48	C	5.61	6.18	–	Yes	28.65	PC	5.24	8.15	–	No	No/28.57
18/1	F L	16.58	C	4.95	2.16	L posterolateral F	No	22.58	C	4.97	2.19	–	Yes	Yes/15.86
18/2	F R	12.87	C	6.03	4.65	–	Yes	18.47	C	3.67	5.27	–	Yes	Yes/3.15
18/3	FT L	–	–	–	–	–	–	18.62	C	3.65	4.50	T L	No	–
19/1	P R	13.13	C	5.72	1.35	–	Yes	31.24	PC	5.34	1.84	–	No	No/27.64
20/1	T pole	15.56	C	3.96	7.52	–	Yes	12.66	C	3.50	7.16	–	Yes	Yes/4.33
21/1	T R	11.68	C	4.26	3.17	L lateral T	Yes	33.92	PC	4.97	0.67	–	No	No/26.85
22/1	FT L	8.26	C	4.16	2.03	–	Yes	48.04	PC	4.36	3.12	L O + R T	No	No/31.75
22/2	T L	25.18	PC	5.39	1.88	–	No	–	–	–	–	–	–	–
23/1	P R	6.23	C	7.18	3.63	–	Yes	14.41	C	3.98	4.65	R middle T	Yes	Yes/10.32
23/2	P R	27.64	PC	4.35	2.12	–	No	31.45	PC	4.11	0.77	–	No	Yes/8.64
24/1	F L	7.65	C	5.18	4.34	–	No	9.17	C	4.50	1.85	Bil Occipital	Yes	Yes/5.93
25/1	T L	24.13	C	3.98	1.05	R lateral T	Yes	21.42	C	4.80	6.54	–	Yes	Yes/5.13
25/2	PO L	18.92	C	5.62	1.54	–	Yes	–	–	–	–	–	–	–
26/1	Bifrontal	13.34	C	6.84	9.25	–	No	33.46	PC	2.98	12.57	L P/R T	No	No/26.21
27/1	FT R	14.42	C	5.34	4.35	–	Yes	10.24	C	4.76	2.64	–	No	Yes/6.95
28/1	F L	7.52	C	3.28	2.46	–	Yes	28.33	PC	5.45	8.34	Bil Occipital	No	No/25.37
29/1	T R	11.62	C	7.26	2.66	R lateral T	Yes	17.46	C	4.13	3.16	–	Yes	Yes/8.62
29/2	FT R	29.85	PC	3.65	2.07	R anterior F	No	31.17	PC	4.80	3.09	L O	No	Yes/10.21
30/1	O R	4.59	C	4.84	2.13	–	Yes	11.09	C	5.12	2.11	–	Yes	Yes/9.64

a *Distance from the center of gravity of the relevant BOLD to the dipole location of the identified component*.

b *Concordance between the BOLD response and the IED location in the proposed analysis*.

c *Concordance between the BOLD response and the IED location in the conventional analysis*.

d *Concordance between the BOLD response in the proposed and the conventional analysis*.

*Bif, bifrontal; Bil, bilateral; C, concordant; D, discordant; F, frontal; FT, frontotemporal; FC, frontocentral; L, left; O, occipital; P, parietal; PC, partially concordant; PO, parieto-occipital; R, right; T, temporal*.

### Concordance Level Evaluation

The results of localization through TCCC determine 35 C, 9 PC, 1 D, and 2 IED studies had no BOLD response. However, 26 C, 15 PC and 3 D were found through the conventional method and 3 EEG-fMRI studies showed no BOLD response. Comparing the two methods based on concordance level evaluation, in 21 cases, the TCCC method confirmed the results of the conventional method.

In three cases, the proposed method was able to provide satisfactory results with one C and 2 PC, while the conventional method was unable to provide results. In 11 IED studies the results of the two methods were not consistent (Table 3, last column); in 10 cases, TCCC improved the results and in only one case (7/2) the conventional method provided better results compared to TCCC (Figure 10).

Since the weakness of the proposed method in the mentioned case is due to the automatic selection of the relevant component (Figure 10, third row), an improved epileptic foci localization may be obtained by manual selection of the component, which leads to results superior to those of the conventional method (Figure 10, fifth row).

## Discussion

Conventional methods for localizing epileptic sources usually consider timings of all IEDs for identifying a seizure zone. However, an IED may be produced by multiple sources located at different brain regions. Therefore, only the voxels of a specific region should be examined for the localization of the seizure generator. Since spikes are frequently detected in a specific area of the electrode domain, it will be helpful to filter out the cortical components that do not show epileptic activity and choose the ones that do. In this study, we proposed a new method that incorporates all temporal information of the identified epileptic sources and avoids being deceived by irrelevant or imperfect information and mistakenly recognizing an unrelated source as a generator of epileptic activity.

The epileptic studies using EEG-fMRI are basically different from the task-based studies, as they consider seizure-related events as opposed to stimulation (41). In the epileptic studies, each source of the large spikes should not be inevitably accepted as an epileptic generator. For example, in focal epilepsy, a large spike detected in the frontal region, which is initiated from the source domain of the parietal lobe is not a valid indicator but the conventional analysis cannot distinguish them, because they use all temporal information of IEDs in one regressor regardless of the corresponding regions. We addressed this problem by adding the spatial information associated with the spike generators to ensure the concordance between the position of the accepted epileptic component and the observed spike in EEG. The concordance of results between various localization methods can improve the reliability of planning surgical resection or interictal EEG (iEEG) electrode placement.

The main inspiration for combining fMRI and EEG measurements comes from the ability to benefit from advantages of each modality. For instance, the EEG-derived activity map alone includes just the weighted sum of electrical activation in the brain with a poor spatial resolution and affected by artifacts, voltage drops, and interference with signals caused by non-epileptic sources. While the EEG signal alone is poorly capable of correctly identifying and localizing the epileptic generators, the EEG components time series associated with epileptic activity can be a consistent indicator. Based on this view, we reveal that hemodynamic correlations of EEG components can detect pathological brain activity. Therefore, simultaneous EEG-fMRI recording with patient's medical record form a ‘golden package’ and extracted component information from scalp EEG that improves the localization of epileptic foci compared to previous methods. This was the case in 90% of the patients in whom the most recent conventional EEG-fMRI analysis was negative, representing that component-related hemodynamic changes could add a more accurate and efficient identification. It is noticeable that in patients who got reasonable results from the conventional analysis, the method of this study also produced similarly concordant results.

In a simultaneous EEG-fMRI acquisition of 34 patients with epilepsy, the TCCC-related BOLD response was observed in all the 30 patients who had IEDs during recording, which makes 90.1% of the whole and is higher performance than that reported in the previous works (18, 42, 43). Also, these responses revealed the epileptic focus in 80% of patients with active EEG (65% of analyzed IEDs), which shows a significant improvement compared to Pittau et al. (18).

Regarding the localization of epileptic generators, Grouiller et al. (44) built an epileptic map by using the spikes detected in the EEG recorded outside the scanner and used it to create a regressor of IEDs from the EEG recorded inside the scanner. Convolving this regressor with an HRF and using it in the GLM analysis revealed concordant BOLD results in 78% (14 of 18) of the patients while this accuracy had a significant rise to 97% using our proposed method. Additional factors also affect the results of analysis, e.g., using higher MRI magnetic field strength which improves the intrasubject reproducibility of EEG-fMRI results (25, 45), using continuous EEG-fMRI instead of spike-triggered which increases the IED-related BOLD response among the patients (46), and using multiple HRFs peaking at 3–9 s after the spike for better localization of the epileptic focus (11, 12, 18).

Drawing on these strategies, our study used multiple HRFs to increase the gain of EEG-fMRI analysis and a high magnetic field MRI scanner to improve the signal-to-noise ratio and reach more informative images. The results showed BOLD responses that were concordant with the spike field in 97% of patients (29 cases out of 30; 74.5% of the analyzed IEDs). This level of concordance which is significantly higher than previous studies (18, 43, 44) is associated with using the component-based approach instead of the linear regressor, multiple HRFs for the fMRI analysis, high-field 3-T scanner for acquiring fMRI data, and effective methods for eliminating artifacts.

However, the definition and evaluation of spatial concordance between the BOLD response and EEG is still to some extent subjective and remains a constant challenge in EEG-fMRI analysis. In our study, the BOLD response is concordant with EEG if the maximum z-value complies with the localization of the EEG spike field. This approach makes the evaluation reliably objective and clinically applicable. All responses have been reported as suggested previously (8, 18). The BOLD responses are more widespread than typical electroclinical findings, due to possible distant or diffuse activations intricate in the epileptic network apart from the focus.

### Concordance Level Scrutiny

Our studies have focused on the use of simultaneous EEG-fMRI for SOZ identification in patients with epilepsy. Since SOZs are best characterized using EEG-fMRI, our TCCC method would be suitable to identify presumed SOZ and evaluate its accuracy by comparing it with the IED-related BOLD activation. The concordance between IED-related BOLD activation and presumed SOZ for different brain structures has not been fully characterized using EEG-fMRI. Besides, seizure types have not been reflected as prominent features for precise identification of the SOZ using EEG-fMRI because of the complex pathophysiology of epileptic cerebral structures. Therefore, there is a fundamental need to quantitatively measure the concordance between IED-related BOLD and presumed SOZ for different brain structures to advance SOZ description. This may provide useful information for surgical guidance and better understanding of the mechanisms underlying seizure generation.

This study examined the distance of maximum BOLD clusters to the location of IED (Figure 10, third, fourth, and fifth rows). The maximum BOLD clusters appeared to be the most clinically relevant responses for all discharge types. From a clinical standpoint, this would assist in identifying the spike-generating network and hence the presumed SOZ.

Concordant BOLD clusters measured up to 25 mm of distance from the center of gravity to the IED contacts while partly concordant clusters measured between 25 and 50 mm of distance when the BOLD cluster is in the same hemisphere. This methodology was used to comprise two confounding factors: (1) electrophysiological activity that does not completely tie with the associated hemodynamic alterations (47) and (2) susceptibility artifact that distorts the BOLD signal up to 20 mm of the electrodes (48). These measures are coherent with an earlier scalp EEG-fMRI study in which high concordance was defined as a distance between the BOLD response and the spike location on scalp EEG between 20 and 40 mm (49). Other EEG-fMRI studies which compared the location of IEDs with the BOLD responses did not describe the concordance as described in the current study; some used any BOLD cluster rather than separating the maximum for evaluation of concordance (28, 44, 50).

Despite the benefits mentioned, it is worth mentioning that the TCCC method heavily depends on accurate component identification. In case, for any reason, the component is identified in other areas, it will exert influence on the results. Although this study proposes a variety of filters to identify appropriate components, there have still been cases in which inaccurate identification performed by the algorithm has led to inaccurate localization (Figure 10, third row). However, the localization improved by the selection of other candidate time series (Figure 10, fifth row).

### Method Limitations

Notwithstanding the mentioned advantages, it should be noted that since we take the time series of the selected components to detect the respective BOLD changes, there will be a plurality to the number of samples of interest, which inevitably makes the proposed method fairly time consuming. The sampling rate for each component is around 250 Hz while the BOLD signal provides one sample per 2.5 s (each TR time). In order to accelerate the process, we have proposed to reduce the sampling rate for the component of interest to the number of BOLD samples. Therefore, we would have invaluable information of the epileptic activity of each generator with respect to the number of BOLD samples.

In this study, although the simultaneous EEG-fMRI method was compared with the epileptic source localization by EEG only and the conventional EEG-fMRI method and its superiority was demonstrated, basically the post-surgical outcome information or the intracranial EEG recording can lead to more reliable results and it more precisely will approve the improvement of the findings.

### Comparison With Other Modalities

Regarding other modalities, PET and SPECT were also used in several studies for the localization of epileptic generators. However, some points need to be considered. For instance, delayed injection in such studies can lead to a misconception of the attained results. Besides, some valuable methods for localizing the epileptic network like interictal FDG PET are not cost-effective while posing risks following radiation exposure.

Generally, although the ictal-based SPECT and PET analyses are suggested and supported by the literature for localizing epileptic foci, their usefulness is limited to revealing regional abnormalities instead of focal epileptic generators (51–54). Besides, our method achieved a BOLD sensitivity of 90% which is higher than those reported for the SPECT and PET studies. However, the obtained specificity might be cooperated by distant BOLD correlations and should be fostered by postsurgical processes. The EEG and MEG source imaging is another promising method that has made incredible development in the number of recorded channels and algorithms to estimate the sources (55–57). The inverse solution of EEG and MEG has reached a sensitivity of around 70% in the study of Knake et al. (58) which is comparable to the EEG-fMRI methods.

### Comparison With Other EEG-fMRI-Based Methods

The classical EEG-fMRI method is an event-related design for fMRI analysis based on the time series of constant amplitude and zero duration or block events with the timing of interictal spikes recorded in the simultaneous EEG (43, 59–61). These interictal spikes are found manually or by an automatic spike detection algorithm based on the spike template acquired from the EEG recorded outside the scanner (9, 34, 44, 62–64). Convolving the time series of events with the standard or patient-specific HRFs (65, 66) makes the base regressor for the GLM analysis to localize BOLD responses as the epileptic generators. The main flaws of the conventional method which calls into question 40–70% of EEG-fMRI studies are: (1) existence of insufficient events during recording; and (2) insignificant BOLD correlation with the observed spikes. One of the proposed solutions for the EEG-fMRI studies with no observed spikes during recording was the fMRI data-driven source identification whose specificity was not particularly promising (67). Therefore, there was a limitation in the cases with detectable spikes for applying similar data-driven approaches premised on spatial independent component analysis of fMRI, and the accomplished results revealed high concordance with those from more conventional methods (38, 67–69).

Also, there are a couple of studies that take continuously fluctuating variables to model epileptic behavior, including continuous electrical imaging (70) which was stated to have enhanced EEG/fMRI by 20%. In addition, the spatial correlation coefficient of the reference topography has been considered in a few studies as a continuously fluctuating parameter to be useful in fMRI analysis (44). This approach can act as a spatial filter analogous to using the strength of a dipole source (71) or the current density that was estimated by electrical source imaging in a specific region (70). Also, some other approaches based on dynamic causal modeling (DCM) (72) and functional connectivity (73) have played a useful role in this regard. This technique despite improving the sensitivity compared to similar studies has not addressed the localization of epileptic generators. Besides, the results of component-based methods have been better than those of the topographic maps even while the presence of detectable spikes during EEG-fMRI recording (70). The source separation methods have been also considered to localize the epileptic focus in the literature (74, 75). In order to identify the epileptic-related components in this method, the EEG signal collected inside the scanner must include spikes or clear focal slowing. The main challenge in such studies is appropriately recognizing the component(s) associated with epileptic activity without using clinical information. We have addressed this challenge by imposing specific conditions on the components along with the convolution of the spike-template obtained from the outside-of-scanner EEG and the candidate ones.

In the study of Bast et al. (34), a new method was proposed to simplify visual detection of spikes in EEG-fMRI premised on spatiotemporal pattern search. For this aim, the principal component analysis (PCA) was applied on a spike template and then its correlation was evaluated with the EEG recorded inside the scanner. The trials with a spatiotemporal correlation above 0.85 were visually evaluated and false-positive identifications manually detached. Although this is a great method to identify the spikes registered inside the scanner, it still uses a traditional linear regressor of temporal information for the GLM analysis.

In total, the mentioned studies provide an overview of the localization of epileptic generators with reasonable accuracy that can be used in real-life applications. Simultaneous EEG-fMRI is a promising combination of temporal and spatial resolution that allows reaching higher prospects for precise localization of epileptic generators in patients with focal epilepsy. In this study, the source domain has been used instead of the sensor field to provide a more accurate recognition of epileptic foci. The results showed that epileptic-related components can be considered as a representative of epileptic foci activity in the GLM analysis and afford clinically precious information even in cases of datasets with inadequate detectable interictal events.

Accurate epileptic foci localization is an essential step in pre-surgical assessments of patients with medically resistant epilepsy. Measuring BOLD changes using EEG-fMRI offers an advanced technique to adequately record abnormal epileptic activities from localized brain regions while capturing related fluctuations in functional brain activities. Further understanding of the epileptogenic zone using IED-related BOLD responses obtained from EEG-fMRI provides a new avenue for clinicians to accurately identify epileptic foci, guide epilepsy surgery, and improve post-surgical results. This study sheds light on the consideration of EEG-fMRI as an indentifer of the epileptic focus, which can be included as part of the clinical assessment for patients with refractory epilepsy.

## Conclusion

This study sets out to provide a realistically estimated pattern of epileptic generators. To do so, we shifted the attention from the electrode domain to the source domain, where we extracted the epilepsy-related components through an ICA analysis. Then, we prioritized these components on the basis of (a) the cross-correlation between the spike-template and the time-series of each component, and (b) their alignment with the complementary physiological information. This would yield a set of ranked components that are most likely contributory to the occurrence of spikes, which can well substitute the simplistic linear regressor in conventional approaches. We went on to convolve the time series of the selected components with HRF and used them in the GLM model and checked if the result was consistent with the physiological EEG observations, if so, we accepted the region as a generator of epileptic activity.

In this study, we have also introduced a new EEG-fMRI method which highlights the correlation between the corresponding BOLD alterations and the spike-related EEG components, which were validated against the gold standard for epileptic generators localization. This approach leads to an increase in the EEG-fMRI yield to non-invasively localize the seizure generators, which is particularly useful in the presurgical evaluation of the patients and implantation of intracranial electrodes, allowing a wider range of patients to consider the option of surgery with more confidence.

In future studies, we intend to apply a new approach for EEG-fMRI integration in the field of epilepsy, which incorporates and tests different models of the transfer function between EEG and BOLD signals, hence allowing better predictions of the hemodynamic changes associated with epileptic activity. This work will therefore provide a contribution to our understanding of the link between EEG and BOLD signals as well as for improving the yield of EEG-fMRI studies in epilepsy (76, 77).

## Data Availability Statement

The datasets presented in this article are not readily available because due to the nature of this research, participants of this study did not agree for their data to be shared publicly. Requests to access the data should be directed to the corresponding author Elias Ebrahimzadeh, e_ebrahimzadeh@ut.ac.ir.

## Ethics Statement

The studies involving human participants were reviewed and approved by Iran University of Medical Sciences. Written informed consent to participate in this study was provided by the participants' legal guardian/next of kin. Written informed consent was obtained from the individuals, and minors' legal guardian/next of kin, for the publication of any potentially identifiable images or data included in this article.

## Author Contributions

EE and HS-Z conceived of the presented idea. EE developed the theory and performed the computations. Material preparation, data collection, and analysis were performed by EE. The first draft of the manuscript was written by EE, MSh, MSe, SS and all authors commented on previous versions of the manuscript. MSh, MSe, and HS-Z verified the analytical methods. The visualization and validation were done by EE, LR, and SS. HS-Z supervised the project. All authors provided critical feedback and helped shape the research, analysis, and manuscript. All authors have read and approved the final manuscript.

## Conflict of Interest

The authors declare that the research was conducted in the absence of any commercial or financial relationships that could be construed as a potential conflict of interest.

## Publisher's Note

All claims expressed in this article are solely those of the authors and do not necessarily represent those of their affiliated organizations, or those of the publisher, the editors and the reviewers. Any product that may be evaluated in this article, or claim that may be made by its manufacturer, is not guaranteed or endorsed by the publisher.

## References

[1] de Boer HM Mula M Sander JW. The global burden and stigma of epilepsy. Epilepsy Behav. (2008) 12:540–546. Available online at: http://ovidsp.ovid.com/ovidweb.cgi?T=JS&PAGE=reference&D=emed8&NEWS=N&AN=2008133201 18280210 10.1016/j.yebeh.2007.12.019

[2] Fisher RS Van Emde Boas W Blume W Elger C Genton P Lee P. Epileptic seizures and epilepsy: definitions proposed by the International League Against Epilepsy (ILAE) and the International Bureau for Epilepsy (IBE). Epilepsia. (2005) 46:470–2. 10.1111/j.0013-9580.2005.66104.x 15816939

[3] Mirbagheri M Hakimi N Ebrahimzadeh E Pourrezaei K Setarehdan SK. Enhancement of optical penetration depth of LED-based NIRS systems by comparing different beam profiles. Biomed Phys Eng Express. (2019) 5:065004. 10.1088/2057-1976/ab42d9/meta

[4] Mirbagheri M Hakimi N Ebrahimzadeh E Setarehdan SK. Quality analysis of heart rate derived from functional near-infrared spectroscopy in stress assessment. Informatics Med Unlocked. (2020) 18:100286. 10.1016/j.imu.2019.100286

[5] Mirbagheri M Hakimi N Ebrahimzadeh E Setarehdan SK. Simulation and *in vivo* investigation of light-emitting diode, near infrared Gaussian beam profiles. J Near Infrared Spectrosc. (2020) 28:37–50. 10.1177/0967033519884209

[6] Rosenow F Lüders H. Presurgical evaluation of epilepsy. Brain. (2001) 124:1683–700. 10.1093/brain/124.9.1683 11522572

[7] Hamer HM Morris HH Mascha EJ Karafa MT Bingaman WE Bej MD. Complications of invasive video-EEG monitoring with subdural grid electrodes. Neurology. (2002) 58:97–103. 10.1212/WNL.58.1.97 11781412

[8] Khoo HM Hao Y von Ellenrieder N Zazubovits N Hall J Olivier A. The hemodynamic response to interictal epileptic discharges localizes the seizure-onset zone. Epilepsia. (2017) 58:811–23. 10.1111/epi.13717 28294306

[9] Tousseyn S Dupont P Robben D Goffin K Sunaert S Van Paesschen W. reliable and time-saving semiautomatic spike-template-based analysis of interictal EEG-fMRI. Epilepsia. (2014) 55:2048–58. 10.1111/epi.12841 25377892

[10] Pedreira C Vaudano AE Thornton RC Chaudhary UJ Vulliemoz S Laufs H. Classification of EEG abnormalities in partial epilepsy with simultaneous EEG-fMRI recordings. Neuroimage. (2014) 99:461–76. 10.1016/j.neuroimage.2014.05.009 24830841

[11] Bagarinao E Maesawa S Ito Y Usui N Natsume J Watanabe H. Detecting sub-second changes in brain activation patterns during interictal epileptic spike using simultaneous EEG-fMRI. Clin Neurophysiol. (2018) 129:377–89. 10.1016/j.clinph.2017.11.018 29288994

[12] Hao Y Khoo HM von Ellenrieder N Zazubovits N Gotman J. DeepIED: an epileptic discharge detector for EEG-fMRI based on deep learning. NeuroImage Clin. (2018) 17:962–75. 10.1016/j.nicl.2017.12.005 29321970 PMC5752096

[13] Morano A Carnì M Casciato S Vaudano AE Fattouch J Fanella M. Ictal EEG/fMRI study of vertiginous seizures. Epilepsy Behav. (2017) 68:51–6. 10.1016/j.yebeh.2016.12.031 28109990

[14] Ebrahimzadeh E Soltanian-Zadeh H Araabi BN. Localization of epileptic focus using simultaneously acquired EEG-FMRI data. Comput Intellig Electric Eng. (2018) 9:15–28. 10.22108/ISEE.2018.111024.1123

[15] Maziero D Velasco TR Salmon CEG Morgan VL. Two-dimensional temporal clustering analysis for patients with epilepsy: detecting epilepsy-related information in EEG-fMRI concordant, discordant and spike-less patients. Brain Topogr. (2018) 31:322–36. 10.1007/s10548-017-0598-3 29022116 PMC5884070

[16] Ives JR Warach S Schmitt F Edelman RR Schomer DL. Monitoring the patient's EEG during echo planar MRI. Electroencephalogr Clin Neurophysiol. (1993) 87:417–20. 10.1016/0013-4694(93)90156-P 7508375

[17] Kay B Szaflarski JP. EEG/fMRI contributions to our understanding of genetic generalized epilepsies. Epilepsy Behav. (2014) 34:129–35. 10.1016/j.yebeh.2014.02.030 24679893 PMC4008674

[18] Pittau F Dubeau F Gotman J. Contribution of EEG/fMRI to the definition of the epileptic focus. Neurology. (2012) 78:1479–87. 10.1212/WNL.0b013e3182553bf7 22539574 PMC3345614

[19] Moeller F Tyvaert L Nguyen DK Levan P Bouthillier A Kobayashi E. EEG-fMRI: Adding to standard evaluations of patients with nonlesional frontal lobe epilepsy. Neurology. (2009) 73:2023–30. 10.1212/WNL.0b013e3181c55d17 19996077 PMC2881856

[20] Bénar CG Gross DW Wang Y Petre V Pike B Dubeau F. The BOLD response to interictal epileptiform discharges. Neuroimage. (2002) 17:1182–92. 10.1006/nimg.2002.1164 12414258

[21] Kobayashi E Bagshaw AP Bénar CG Aghakhani Y Andermann F Dubeau F. Temporal and extratemporal BOLD responses to temporal lobe interictal spikes. Epilepsia. (2006) 47:343–54. 10.1111/j.1528-1167.2006.00427.x 16499759

[22] Thornton R Laufs H Rodionov R Cannadathu S Carmichael DW Vulliemoz S. EEG correlated functional MRI and postoperative outcome in focal epilepsy. J Neurol Neurosurg Psychiatry. (2010) 81:922–7. 10.1136/jnnp.2009.196253 20547617

[23] Zijlmans M Huiskamp G Hersevoort M Seppenwoolde JH Van Huffelen AC Leijten FSS. EEG-fMRI in the preoperative work-up for epilepsy surgery. Brain. (2007) 130:2343–53. 10.1093/brain/awm141 17586868

[24] Noachtar S Borggraefe I. Epilepsy surgery: a critical review. Epilepsy Behav. (2009) 15:66–72. 10.1016/j.yebeh.2009.02.028 19236942

[25] Gholipour T Moeller F Pittau F Dubeau F Gotman J. Reproducibility of interictal EEG-fMRI results in patients with epilepsy. Epilepsia. (2011) 52:433–42. 10.1111/j.1528-1167.2010.02768.x 21054351 PMC3792085

[26] Ridley B Wirsich J Bettus G Rodionov R Murta T Chaudhary U. Simultaneous intracranial EEG-fMRI shows inter-modality correlation in time-resolved connectivity within normal areas but not within epileptic regions. Brain Topogr. (2017) 30:639–55. 10.1007/s10548-017-0551-5 28194612

[27] Beers CA Williams RJ Gaxiola-Valdez I Pittman DJ Kang AT Aghakhani Y. Patient specific hemodynamic response functions associated with interictal discharges recorded via simultaneous intracranial EEG-fMRI. Hum Brain Mapp. (2015) 36:5252–64. 10.1002/hbm.23008 26417648 PMC6869833

[28] Vulliemoz S Carmichael DW Rosenkranz K Diehl B Rodionov R Walker MC. Simultaneous intracranial EEG and fMRI of interictal epileptic discharges in humans. Neuroimage. (2011) 54:182–90. 10.1016/j.neuroimage.2010.08.004 20708083

[29] Ebrahimzadeh E Soltanian-Zadeh H Nadjar Araabi B Hashemi Fesharaki SS Mehvari Habibabadi J. Component-related BOLD response to localize epileptic focus using simultaneous EEG-fMRI recordings at 3T. J Neurosci Methods. (2019) 322:34–49. 10.1016/j.jneumeth.2019.04.010 31026487

[30] Seraji M Mohebbi M Safari A Krekelberg B. Multiple sclerosis reduces synchrony of the magnocellular pathway. PLoS ONE. (2021) 16:e0255324. 10.1371/JOURNAL.PONE.0255324 34437558 PMC8389379

[31] Raeisi K Mohebbi M Khazaei M Seraji M Yoonessi A. Phase-synchrony evaluation of EEG signals for Multiple Sclerosis diagnosis based on bivariate empirical mode decomposition during a visual task. Comput Biol Med. (2020) 117:103596. 10.1016/J.COMPBIOMED.2019.103596 32072973

[32] Ebrahimzadeh E Asgarinejad M Saliminia S Ashoori S Seraji M. Predicting clinical response to transcranial magnetic stimulation in major depression using time-frequency EEG signal processing. Biomed Eng. (2021). 10.4015/S1016237221500484

[33] Ebrahimzadeh E Shams M Rahimpour Jounghani A Fayaz F Mirbagheri M Hakimi N. Localizing confined epileptic foci in patients with an unclear focus or presumed multifocality using a component-based EEG-fMRI method. Cogn Neurodyn. (2020) 5:1–16. 10.1007/s11571-020-09614-5 33854640 PMC7969677

[34] Bast T Oezkan O Rona S Stippich C Seitz A Rupp A. and MEG source analysis of single and averaged interictal spikes reveals intrinsic epileptogenicity in focal cortical dysplasia. Epilepsia. (2004) 45:621–31. 10.1111/j.0013-9580.2004.56503.x 15144427

[35] LeVan P Tyvaert L Moeller F Gotman J. Independent component analysis reveals dynamic ictal BOLD responses in EEG-fMRI data from focal epilepsy patients. Neuroimage. (2010) 49:366–78. 10.1016/j.neuroimage.2009.07.064 19647798 PMC3779215

[36] Ebrahimzadeh E Shams M Fayaz F Rajabion L Mirbagheri M Nadjar Araabi B. Quantitative determination of concordance in localizing epileptic focus by component-based EEG-fMRI. Comput Methods Programs Biomed. (2019) 177:231–41. 10.1016/j.cmpb.2019.06.003 31319952

[37] Ebrahimzadeh E Amoozegar S Asgarinejad M Dolatabad MR Bagheri M Zangeneh Soroush M. Simultaneous EEG-fMRI: a multimodality approach to localize the seizure onset zone in patients with epilepsy. Int J Biol Med. (2019) 1:130–9. 10.36811/ijbm.2019.110017

[38] LeVan P Gotman J. Independent component analysis as a model-free approach for the detection of BOLD changes related to epileptic spikes: a simulation study. Hum Brain Mapp. (2009) 30:2021–31. 10.1002/hbm.20647 18726909 PMC3792083

[39] Worsley KJ Liao CH Aston J Petre V Duncan GH Morales F. General statistical analysis for fMRI data. Neuroimage. (2002) 15:1–15. 10.11253/ninchishinkeikagaku1999.3.91 11771969

[40] Sadjadi SM Ebrahimzadeh E Shams M Seraji M Soltanian-Zadeh H. Localization of epileptic foci based on simultaneous EEG–fMRI data. Front Neurol. (2021) 12:472. 10.3389/fneur.2021.645594 33986718 PMC8110922

[41] Müller-Bardorff M Bruchmann M Mothes-Lasch M Zwitserlood P Schlossmacher I Hofmann D. Early brain responses to affective faces: A simultaneous EEG-fMRI study. Neuroimage. (2018) 178:660–7. 10.1016/j.neuroimage.2018.05.081 29864521

[42] Al-Asmi A Bénar CG Gross DW Agha Khani Y Andermann F Pike B. fMRI Activation in continuous and spike-triggered EEG-fMRI studies of epileptic spikes. Epilepsia. (2003) 44:1328–39. 10.1046/j.1528-1157.2003.01003.x 14510827

[43] Salek-Haddadi A Diehl B Hamandi K Merschhemke M Liston A Friston K. Hemodynamic correlates of epileptiform discharges: an EEG-fMRI study of 63 patients with focal epilepsy. Brain Res. (2006) 1088:148–66. 10.1016/j.brainres.2006.02.098 16678803

[44] Grouiller F Thornton RC Groening K Spinelli L Duncan JS Schaller K. With or without spikes: localization of focal epileptic activity by simultaneous electroencephalography and functional magnetic resonance imaging. Brain. (2011) 134:2867–86. 10.1093/brain/awr156 21752790 PMC3656675

[45] Krüger G Kastrup A. Glover GH. Neuroimaging at 15 T and 30 T: comparison of oxygenation-sensitive magnetic resonance imaging. Magn Reson Med. (2001) 45:595–604. 10.1002/mrm.1081 11283987

[46] Lemieux L Mulert C. EEG–fMRI Physiological Basis, Technique and Applications. Berlin: Spriinger. (2009).

[47] Disbrow EA Slutsky DA Roberts TPL. Krubitzer LA. Functional MRI at 15 tesla: A comparison of the blood oxygenation level-dependent signal and electrophysiology. Proc Natl Acad Sci USA. (2000) 97:9718–23. 10.1073/pnas.170205497 10931954 PMC16931

[48] Boucousis SM Beers CA Cunningham CJB Gaxiola-Valdez I Pittman DJ Goodyear BG. Feasibility of an intracranial EEG-fMRI protocol at 3T: risk assessment and image quality. Neuroimage. (2012) 63:1237–48. 10.1016/j.neuroimage.2012.08.008 22902923

[49] Bénar CG Grova C Kobayashi E Bagshaw AP Aghakhani Y Dubeau F. EEG-fMRI of epileptic spikes: concordance with EEG source localization and intracranial EEG. Neuroimage. (2006) 30:1161–70. 10.1016/j.neuroimage.2005.11.008 16413798

[50] Aghakhani Y Beers CA Pittman DJ Gaxiola-Valdez I Goodyear BG Federico P. Co-localization between the BOLD response and epileptiform discharges recorded by simultaneous intracranial EEG-fMRI at 3 T. NeuroImage Clin. (2015) 7:755–63. 10.1016/j.nicl.2015.03.002 25844327 PMC4375646

[51] Wetjen NM Cascino GD Fessler AJ So EL Buchhalter JR Mullan BP. Subtraction ictal single-photon emission computed tomography coregistered to magnetic resonance imaging in evaluating the need for repeated epilepsy surgery. J Neurosurg. (2006) 105:71–6. 10.3171/jns.2006.105.1.71 16874891

[52] Willmann O Wennberg R May T Woermann FG Pohlmann-Eden B. The contribution of 18F-FDG PET in preoperative epilepsy surgery evaluation for patients with temporal lobe epilepsy. A meta-analysis. Seizure. (2007) 16:509–20. 10.1016/j.seizure.2007.04.001 17532231

[53] O'Brien TJ So EL Mullan BP Hauser MF Brinkmann BH Bohnen NI. Subtraction ictal SPECT co-registered to MRI improves clinical usefulness of SPECT in localizing the surgical seizure focus. Neurology. (1998) 50:445–54. 10.1212/WNL.50.2.445 9484370

[54] O'Brien TJ So EL Mullan BP Hauser MF Brinkmann BH Jack CR. Subtraction SPECT co-registered to MRI improves postictal SPECT localization of seizure loci. Neurology. (1999) 52:137–46. 10.1212/wnl.52.1.137 9921861

[55] Michel CM Lantz G Spinelli L De Peralta RG Landis T Seeck M. 128-Channel EEG source imaging in epilepsy: clinical yield and localization precision. J Clin Neurophysiol. (2004) 21:71–83. 10.1097/00004691-200403000-00001 15284597

[56] Michel CM Murray MM Lantz G Gonzalez S Spinelli L Grave De Peralta R. EEG source imaging. Clin Neurophysiol. (2004) 115:2195–222. 10.1016/j.clinph.2004.06.001 15351361

[57] Shibasaki H Ikeda A Nagamine T. Use of magnetoencephalography in the presurgical evaluation of epilepsy patients. Clin Neurophysiol. (2007) 118:1438–48. 10.1016/j.clinph.2007.03.002 17452007

[58] Knake S Halgren E Shiraishi H Hara K Hamer HM Grant PE. The value of multichannel MEG and EEG in the presurgical evaluation of 70 epilepsy patients. Epilepsy Res. (2006) 69:80–6. 10.1016/j.eplepsyres.2006.01.001 16516443

[59] Lemieux L Salek-Haddadi A Josephs O Allen P Toms N Scott C. Event-related fMRI with simultaneous and continuous EEG: description of the method and initial case report. Neuroimage. (2001) 14:780–7. 10.1006/nimg.2001.0853 11506550

[60] Gotman J. Epileptic networks studied with EEG-fMRI. Epilepsia. (2008) 49:42–51. 10.1111/j.1528-1167.2008.01509.x 18304255 PMC3792078

[61] Bagshaw AP Hawco C Bénar CG Kobayashi E Aghakhani Y Dubeau F. Analysis of the EEG-fMRI response to prolonged bursts of interictal epileptiform activity. Neuroimage. (2005) 24:1099–112. 10.1016/j.neuroimage.2004.10.010 15670687

[62] Ebrahimzadeh E Shams M Rahimpour Jopungha A Fayaz F Mirbagheri M Hakimi N. Epilepsy presurgical evaluation of patients with complex source localization by a novel component-based EEG-fMRI approach. Iran J Radiol. (2019) 16:99134. 10.5812/iranjradiol.99134

[63] Ebrahimzadeh E Soltanian-Zadeh H Araabi BN Fesharaki SSH Habibabadi JM. Localizing epileptic focus through simultaneous EEG-fMRI recording and automated detection of IED from inside-scanner EEG. Iran J Biomed Eng. (2019) 13:135–45. 10.22041/ijbme.2019.103479.1447

[64] Ebrahimzadeh E Soltanian-Zadeh H Araabi BN Fesharaki SSH Habibabadi JM. Localizing epileptic focus through simultaneous EEG-fMRI recording and automated detection of interictal epileptiform discharges (IED) from EEG in inside MRI. in 25th National and 3rd International Iranian Conference on Biomedical Engineering (ICBME 2018), 1–6.

[65] van Houdt PJ de Munck JC Zijlmans M Huiskamp G Leijten FSS Boon PAJM. Comparison of analytical strategies for EEG-correlated fMRI data in patients with epilepsy. Magn Reson Imaging. (2010) 28:1078–86. 10.1016/j.mri.2010.03.022 20471191

[66] Kang JK Bénar CG Al-Asmi A Khani YA Pike GB Dubeau F. Using patient-specific hemodynamic response functions in combined EEG-fMRI studies in epilepsy. Neuroimage. (2003) 20:1162–70. 10.1016/S1053-8119(03)00290-8 14568485

[67] Rodionov R De Martino F Laufs H Carmichael DW Formisano E Walker M. Independent component analysis of interictal fMRI in focal epilepsy: comparison with general linear model-based EEG-correlated fMRI. Neuroimage. (2007) 38:488–500. 10.1016/j.neuroimage.2007.08.003 17889566

[68] Thornton RC Rodionov R Laufs H Vulliemoz S Vaudano A Carmichael D. Imaging haemodynamic changes related to seizures: comparison of EEG-based general linear model, independent component analysis of fMRI and intracranial EEG. Neuroimage. (2010) 53:196–205. 10.1016/j.neuroimage.2010.05.064 20570736

[69] Moeller F LeVan P Gotman J. Independent component analysis (ICA) of generalized spike wave discharges in fMRI: comparison with general linear model-based EEG-fMRI. Hum Brain Mapp. (2011) 32:209–17. 10.1002/hbm.21010 20336659 PMC3753294

[70] Vulliemoz S Rodionov R Carmichael DW Thornton R Guye M Lhatoo SD. Continuous EEG source imaging enhances analysis of EEG-fMRI in focal epilepsy. Neuroimage. (2010) 49:3219–29. 10.1016/j.neuroimage.2009.11.055 19948231

[71] Liston AD De Munck JC Hamandi K Laufs H Ossenblok P Duncan JS. Analysis of EEG-fMRI data in focal epilepsy based on automated spike classification and Signal Space Projection. Neuroimage. (2006) 31:1015–24. 10.1016/j.neuroimage.2006.01.040 16545967

[72] Hamandi K Powell HWR Laufs H Symms MR Barker GJ Parker GJM. Combined EEG-fMRI and tractography to visualise propagation of epileptic activity. J Neurol Neurosurg Psychiatry. (2008) 79:594–7. 10.1136/jnnp.2007.125401 18096681 PMC2571962

[73] Murta T Figueiredo P Leal A. EEG-fMRI measures of functional brain connectivity in epilepsy. in 1st Portuguese Meeting in Biomedical Engineering, ENBENG 2011 10.1109/ENBENG.2011.6026094

[74] Jann K Wiest R Hauf M Meyer K Boesch C Mathis J Schroth G Dierks T Koenig T. BOLD correlates of continuously fluctuating epileptic activity isolated by independent component analysis. Neuroimage. (2008) 42:635–648. Available online at: http://ovidsp.ovid.com/ovidweb.cgi?T=JS&PAGE=reference&D=emed8&NEWS=N&AN=2008357364 18585061 10.1016/j.neuroimage.2008.05.001

[75] Marques JP Rebola J Figueiredo P Pinto A Sales F Castelo-Branco M. decomposition of EEG signal for fMRI processing in epilepsy. Hum Brain Mapp. (2009) 30:2986–96. 10.1002/hbm.20723 19172633 PMC6870975

[76] Abreu R Leal A Lopes da. Silva F, Figueiredo P. EEG synchronization measures predict epilepsy-related BOLD-fMRI fluctuations better than commonly used univariate metrics. Clin Neurophysiol. (2018) 129:618–35. 10.1016/j.clinph.2017.12.038 29414405

[77] Leite M Leal A Figueiredo P. Transfer function between EEG and BOLD signals of epileptic activity. Front Neurol. (2013) 4 JAN: 10.3389/fneur.2013.00001 23355832 PMC3554836

